# Xuanfei Baidu Decoction and Its Active Component Glycyrrhizic Acid Ameliorate Insulin Resistance in Type 2 Diabetes via JNK/JAK/STAT Axis Inhibition

**DOI:** 10.3390/biology15211975

**Published:** 2026-11-02

**Authors:** Nana He, Xinyi Li, Lifeng Han, Liming Wang, Yidong Xu, Shushen Sun, Siqi Guo, Yiwen Wang

**Affiliations:** 1 School of Pharmaceutical Science and Technology, Faculty of Medicine, Tianjin University, Tianjin 300072, China; 2 State Key Laboratory of Component-Based Chinese Medicine, Tianjin Key Laboratory of TCM Chemistry and Analysis, Instrumental Analysis & Research Center, Tianjin University of Traditional Chinese Medicine, Tianjin 301617, China; 3 Department of Gastroenterology, Tianjin University Jinnan Hospital, Tianjin 300350, China

**Keywords:** type 2 diabetes mellitus, Xuanfei Baidu decoction, *Drosophila melanogaster*, JNK signaling pathway, JAK/STAT signaling pathway, glycyrrhizic acid, insulin resistance, network pharmacology

## Abstract

Background: Type 2 diabetes mellitus (T2DM) is driven by insulin resistance and chronic inflammation, yet current single-target therapies inadequately address these interconnected pathologies. Xuanfei Baidu decoction (XFBD), originally developed for COVID-19, is known to inhibit JNK and JAK/STAT pathways and may offer a multi-target strategy. Methods: A high sugar diet (HSD) induced *Drosophila* T2DM model was treated with XFBD or its active component glycyrrhizic acid (GA). Phenotypic assays, metabolomics, 16S rRNA sequencing, qPCR, transgenic reporter flies, network pharmacology, and computational docking/dynamics simulations were performed. Results: XFBD dose-dependently alleviated HSD-induced developmental delay, hyperglycemia, insulin resistance (restored dilp2/3/5, InR, 4EBP expression and AKT phosphorylation), glycolipid disorders, and oxidative stress. Metabolomics identified 79 reversed metabolites enriched in T2DM-relevant pathways (D-amino acid, BCAA, TCA cycle). XFBD suppressed aberrant JNK and JAK/STAT activation, independent of gut microbiota modulation. GA recapitulated these effects in vivo. Conclusions: XFBD and GA ameliorate insulin resistance and metabolic dysregulation via JNK/JAK-STAT axis inhibition, providing a preclinical rationale for their therapeutic application in T2DM and related metabolic syndrome outcomes.

## Simple Summary

Type 2 diabetes is a growing global health problem in which the body becomes resistant to the effects of insulin, often accompanied by chronic low-grade inflammation. Current treatments typically target a single pathway, leaving many patients with inadequate control. Xuanfei Baidu Decoction (XFBD) is a traditional Chinese herbal formula originally developed during the COVID-19 pandemic; it has since been shown to have broad anti-inflammatory effects, but whether it can improve insulin resistance has been unclear. In this study, we used fruit flies fed a high-sugar diet as a simple and rapid model of type 2 diabetes. We found that XFBD dose-dependently reversed the diabetic features—including elevated blood sugar, delayed development, and disrupted fat. XFBD worked by calming down two major inflammatory signaling pathways (JNK and JAK/STAT), which in turn restored normal insulin signaling. This effect did not depend on changes in gut bacteria. We further identified glycyrrhizic acid (GA), a major component of the formula, as one of its key active ingredients that reproduced the benefits on its own. These findings suggest that XFBD and GA offer a promising multi-target strategy for treating insulin resistance and related metabolic disorders, and provide a rationale for further testing in mammalian models and clinical studies.

## 1. Introduction

Type 2 diabetes mellitus (T2DM) accounts for approximately 90–95% of all diabetes cases, affecting 537 million adults globally, and exceeding 700 million by 2045 [1,2,3,4]. T2DM frequently co-occurs with obesity and dyslipidemia in metabolic syndrome (MetS), sharing core pathophysiological roots in insulin resistance and chronic low-grade inflammation [5,6,7,8,9,10]. Conventional T2DM pharmacotherapy predominantly target single nodes of glucose homeostasis, often failing to address the underlying inflammatory pathology or broader metabolic dysregulation of MetS, while being associated with notable long-term side effects [11,12,13].

The c-Jun N-terminal kinase (JNK) and the Janus kinase/signal transducer and activator of transcription (JAK/STAT) pathways serve as two critical nodes connecting inflammatory stimuli to impaired insulin signaling [14,15,16,17,18]. Under high-calorie stress, aberrant JNK activation in adipose tissue directly impairs insulin signaling via inhibitory serine phosphorylation of insulin receptor substrate-1 (IRS-1), thereby disrupting downstream PI3K-AKT signal transduction [19,20,21]. Concurrently, JNK-driven secretion of IL-6 and other cytokines activates the JAK/STAT pathway, which upregulates suppressors of cytokine signaling (SOCS) proteins that competitively inhibit insulin receptor tyrosine kinase activity and target IRS proteins for ubiquitin-mediated degradation [22,23,24]. Notably, these two pathways form a positive feedback loop: STAT-mediated transcriptional upregulation of pro-inflammatory genes also amplifies JNK activation, creating a self-sustaining cycle that perpetuates insulin resistance and metabolic dysfunction [25,26,27]. Accordingly, simultaneously targeting both pathways to break this cycle may achieve superior efficacy over single-pathway inhibition, offering potential to ameliorate the full spectrum of metabolic abnormalities.

With multi-component, multi-target pharmacology, TCM formulas offer conceptually attractive therapeutic strategies for complex metabolic diseases like T2DM, where multiple interconnected pathogenic pathways converge to drive disease progression [28,29,30,31]. Xuanfei Baidu decoction (XFBD), an 11-herb formula (Ephedrae Herba (6 g), Armeniacae Semen Amarum (9 g), Gypsum Fibrosum (15 g), Coicis Semen (30 g), Atractylodis Rhizoma (10 g), Pogostemonis Herba (10 g), Artemisiae Annuae Herba (12 g), Polygoni Cuspidati Rhizoma et Radix (15 g), Verbenae Herba (15 g), Citri Reticulatae Pericarpium (6 g), and Glycyrrhizae Radix et Rhizoma (6 g)), was originally developed for COVID-19 and has demonstrated robust clinical efficacy in reducing systemic inflammation and accelerating symptom resolution [32,33,34]. In TCM theory, XFBD acts by dispersing lung pathogens, clearing heat and resolving dampness, and fortifying the spleen [33]. T2DM is broadly categorized in TCM as the “Xiaoke” category, with spleen deficiency, dampness retention and damp-heat accumulation as core pathological patterns. XFBD’s spleen-fortifying, heat-clearing and dampness-resolving actions align theoretically with these features, supporting exploration of its metabolic potential [35]. Studies in *Drosophila* and mouse models have confirmed XFBD’s anti-inflammatory and immunomodulatory activities, via inhibition of NF-κB, JNK, JAK/STAT, and MAPK signaling pathways, as well as modulation of gut microbiota homeostasis in acute inflammatory disease settings [32,34,36,37,38,39]. Beyond demonstrating that XFBD alleviates severe COVID-19 via the gut-lung axis, these findings have broadened its candidate indications to inflammatory bowel disease (IBD). Given that JNK and JAK/STAT signaling, as well as gut microbiota homeostasis, represent key drivers of both T2DM and MetS pathogenesis, XFBD holds theoretical therapeutic potential for glucolipid metabolic disorders. However, whether XFBD exerts a protective effect against T2DM and associated metabolic dysregulation, and which mechanism predominates in the metabolic context, remains largely uncharacterized.

The *Drosophila melanogaster* model offers unique advantages for metabolic disease research and drug discovery [37,40]. In particular, it enables rapid, low-cost, and high-throughput establishment of insulin resistance models that retain high physiological relevance, outperforming mammalian systems in screening efficiency [41,42,43]. Mechanistically, a high-sucrose diet (HSD) alone is sufficient to induce gut microbiota dysbiosis and trigger sustained activation of JNK and JAK/STAT inflammatory pathways in wild-type flies, collectively driving systemic insulin resistance and hallmark T2DM phenotypes including hyperglycemia, lipid accumulation, and oxidative stress [44,45]. Notably, this HSD-induced inflammatory cascade and metabolic dysfunction in *Drosophila* closely recapitulates the pathogenesis of diet-induced insulin resistance in mammals, with high evolutionary conservation of core signaling pathways [46,47,48].

Given the well-documented anti-inflammatory properties of XFBD and the central role of the JNK-JAK/STAT inflammatory axis in metabolic disorders [45], the present study aimed to investigate, for the first time in the *Drosophila* model, whether XFBD ameliorates HSD-induced insulin resistance via modulating gut microbiota composition and/or suppressing JNK and JAK/STAT inflammatory pathway activity.

## 2. Materials and Methods

### 2.1. Drosophila Culture and Insulin Resistance Model

The following *Drosophila melanogaster* strains were used: wild-type Dijon2000 (obtained from France by Dr. Jean-Francois Ferveur [49]); upd3-Gal4 > UAS-GFP (THU0198, Tsinghua University Fly Center) and 10 × STAT-GFP (donated by Professor Song’s laboratory at Wuhan University [50]). For the genetic validation experiments, UAS-*upd3* (TH13268.S, Tsinghua University Fly Center) and UAS-*bsk*^DN^ (TH14716.S, Tsinghua University Fly Center) flies were crossed with the appropriate Gal4 (Cg-Gal4, BDSC_7011, Bloomington Drosophila Stock Center; *Myo1A*-Gal4, donated by Professor Song’s laboratory at Wuhan University) driver to generate *upd3* overexpression and dominant-negative JNK expression progeny, respectively. All strains were maintained at 25 °C with 60% relative humidity under a 12-h light/dark cycle. The normal diet (ND) consisted of 1% agar, 8% yeast, 2% yeast extract, and 5% sucrose (*w*/*v*). The HSD was prepared by increasing sucrose from 5% to 48% (*w*/*v*) as described by Y. Li et al. [45]. XFBD (provided by Tianjin Modern TCM Innovation Center) was supplemented into the HSD medium at 0.3%, 1%, and 3% (*w*/*v*). Metformin (Met, 0.001% *w*/*v*, CAS: 657-24-9, MCE, Shanghai, China) served as a positive control. Glycyrrhizic acid (GA, purity ≥98%, CAS: 1405-86-3, MCE, Shanghai, China) was tested at 0.2, 1, 2, and 10 μg/mL to establish a full dose-response relationship. For pharmacological JAK/STAT inhibition, flies were maintained on HSD medium supplemented with 25 μM methotrexate (MTX, CAS: 2791417-66-6, MCE, China) for 7 days. All experiments were performed on female flies aged 3–5 days to exclude sex-related metabolic variation.

### 2.2. Phenotypic Assessment

#### 2.2.1. Pupariation and Eclosion Rates

Developmental viability was assessed by determining pupariation and eclosion rates. Mated female flies (3–5 days post-eclosion, 15 per replicate) were transferred to experimental diets for oviposition, with five biological replicates per group. After a 24-h oviposition period, flies were removed from the vials. Puparia and emerged adults were scored daily. For each replicate, the absolute rate was first calculated, then normalized to the mean rate of the control group, with the control set to 100%.

#### 2.2.2. Pupal Size and Wing D Region Area

For pupal size, 20 pupae per group were randomly selected, photographed using a digital microscope camera (cnoptec sz680), and length (L) and maximum width (W) were measured with AJ-VERT software x64, 6.0.20694.20220417. Pupal volume was calculated as 4/3π(L/2)(W/2)^2^ (L: length; W: width) [51]. For wing morphometry, newly eclosed adult flies were collected, and the left wing of each fly was dissected, flat-mounted on a glass slide, and imaged under a stereomicroscope. The area of the wing D region was quantified with the same AJ-VERT software.

#### 2.2.3. Hemolymph Glucose and Trehalose Levels

Hemolymph glucose and trehalose concentrations were measured using a D-glucose (glucose oxidase method) kit (CAS: A154-1-1, Naniing Jiancheng, Nanjin, China). Female flies reared on experimental media for 7 days were collected (*n* = 40 per replicate, ≥3 biological replicates per group). Hemolymph was harvested via thoracic puncture on ice, and centrifuged at 15,000× *g* for 15 min at 4 °C. A total of 1 μL of hemolymph was diluted in 9 μL PBS, heat-inactivated at 70 °C for 5 min, and centrifuged at 14,000× *g* for 10 min at 4 °C. For free glucose measurement, 1 μL of supernatant was mixed with 100 μL glucose chromogenic reagent and incubated at 37 °C for 30 min; absorbance was read at 505 nm. Subsequently, 20 μL trehalase was added to the reaction system and incubated at 37 °C for a further 30 min to hydrolyze trehalose. Trehalose content was calculated from the absorbance difference between readings after the second incubation minus that after the first.

#### 2.2.4. Glycogen Staining

Glycogen deposition in the adult fat body was visualized by periodic acid-Schiff (PAS) staining. Fat bodies dissected from female flies were mounted on glass slides in PBS, air-dried at room temperature, and fixed with PAS fixative for 15 min. After rinsing, the slides were air-dried again, then oxidized for 15 min, sequentially rinsed with running water and distilled water, and re-dried. Slides were stained with Schiff’s reagent for 15 min in the dark, rinsed under running water for 5 min, and imaged under a light microscope.

#### 2.2.5. Body Weight, Water and Lipid Contents

Twenty female flies per replicate were weighed. to obtain initial body weight. Flies wrapped in aluminum foil were dried at 70 °C for 4 h, and water content was calculated as the difference between wet and dry weight. For lipid extraction, total lipids were extracted by immersion in 1 mL anhydrous diethyl ether for 12 h at room temperature. After ether removal, samples were re-dried at 70 °C to constant weight, and lipid content was derived from the weight difference before and after ether extraction.

### 2.3. DHE Staining

Intestinal reactive oxygen species (ROS) levels were measured by dihydroethidium (DHE) staining (CAS:ID3560, Solarbio, Beijing, China). Intestines were dissected from 20 flies per group in ice-cold PBS, then incubated with 45 μM DHE for 5 min at 37 °C in the dark. After three 5-min washes with PBS, samples were counterstained with DAPI (10 min), followed by three washes with PBS (10 min each). Intestines were mounted on glass slides, coverslips applied, and sealed with nail polish. Fluorescence images were captured under an upright fluorescence microscope, and fluorescence intensity was quantified using ImageJ 1.54f.

### 2.4. GFP Fluorescence Imaging

To monitor JAK/STAT signaling activity and upd3 expression ex vivo, 10 × STAT-GFP and *upd3*-Gal4 > UAS-GFP reporter flies were reared on experimental diets for 7 days. Intestines were dissected in ice-cold PBS and fixed in 4% paraformaldehyde (PFA) for 5 min at room temperature. After three 10 min PBS washes, tissues were counterstained with DAPI (10 min, dark) and washed again (three 10 min PBS washes). Stained intestines were mounted on glass slides and sealed with nail polish. Fluorescence images were acquired using an upright fluorescence microscope (ECLIPSE 80i, NIKON CORPORATION, Tokyo, Japan) with identical exposure times and gain settings across all samples. GFP signal intensity was quantified using ImageJ, and the mean fluorescence intensity per intestinal area was calculated after background subtraction.

### 2.5. Non-Targeted Metabolomics

Flies reared on experimental diets for 7 days were collected (8 biological replicates per group; 20 flies per replicate). Samples were snap-frozen in liquid nitrogen and stored at −80 °C until analysis. Metabolomic profiling was conducted at the Instrumental Analysis and Research Center, Tianjin University of Traditional Chinese Medicine. The metabolomics data have been deposited in the MetaboLights repository under the accession number REQ20260927224356.

### 2.6. Gut Microbiota Analysis

Flies reared on each experimental diet for 7 days were collected at 0.3 g per sample (4 biological replicates per group). After snap-freezing in liquid nitrogen, the samples were stored at −80 °C until sequencing. 16S rRNA gene sequencing was performed by Majorbio Bio-Pharm Technology Co., Ltd. (Shanghai, China), and data were analyzed using the BMK Cloud platform. The 16S rRNA sequencing data have been deposited in the NCBI Sequence Read Archive (SRA) under the submission number SUB16514444.

### 2.7. RT-qPCR

Total RNA was extracted (10 female flies per replicate; 4 biological replicates per group) using TRIzol reagent (Solarbio, Cat: R1100, Beijing, China). RNA concentration and purity were determined using a NanoDrop One spectrophotometer. RNA was reverse transcribed into cDNA using the Evo M-MLV Reverse Transcription Kit (Accurate Biology, Cat:AG11705, Tianjin, China). Quantitative real-time PCR was performed on a Q2000 Real-Time PCR Detection System using SYBR Green Pro Taq HS (Accurate Biology, Cat:AG11701, Tianjin, China) with the following cycling conditions: 95 °C for 30 s, followed by 40 cycles of 95 °C for 5 s and 60 °C for 30 s. Primer sequences are provided in Supplementary Table S1. Target gene expression was normalized to *rp49* (reference gene) using the 2^−ΔΔCt^ method.

### 2.8. Network Pharmacology

Active components of XFBD were identified based on previous UPLC-Q-TOF/MS results from our previous work [34], importantly, the XFBD preparation used in the present study is from the same batch; their SMILES were retrieved from PubChem and imported into SwissTargetPrediction for target prediction (probability > 0). T2DM-related targets were retrieved from GeneCards, OMIM, and TTD databases, filtered per each database’s criteria, merged, and deduplicated. The herb-compound-target network was constructed using Cytoscape 3.10.2, with nodes representing herbs, compounds, or targets and edges indicating interactions. Intersecting targets between compound-derived and disease-derived sets were identified using Venny 2.1.0. These overlapping targets were submitted to the STRING database (species: Homo sapiens; interaction score ≥ 0.4) to construct a PPI network; isolated nodes were removed, and the network was visualized in Cytoscape 3.10.2. Core targets were screened via topological analysis using the Centiscape 2.2 plugin, with targets exceeding the median values of degree, closeness, and betweenness centrality retained. Finally, GO (BP, CC, MF) and KEGG pathway enrichment analyses were performed using DAVID (https://davidbioinformatics.nih.gov/); the top 10 GO terms (per category) and top 20 KEGG pathways were selected by gene ratio and visualized as bar charts and bubble charts, respectively, using the bioinformatics platform (www.bioinformatics.com.cn).

### 2.9. Molecular Docking and Molecular Dynamics Simulation

To validate the network pharmacology predictions, molecular docking was performed using AutoDockTools-1.5.7 to assess binding affinities between core targets (STAT3 and JUN) and their corresponding active components. Ligand 3D structures were retrieved from PubChem in SDF format and converted to PDB format using OpenBabel 3.1.0; target protein structures were downloaded from RCSB PDB (https://www.rcsb.org). Binding poses and interactions were visualized with PyMOL 3.1.9. To further evaluate complex stability, molecular dynamics (MD) simulations were carried out using GROMACS 2026.4. Each protein ligand complex was subjected to energy minimization, followed by NVT (constant volume and temperature) and NPT (constant pressure and temperature) equilibration, and finally a 100 ns production MD simulation. Post-simulation analyses included RMSD, Rg, RMSF, and SASA to assess structural stability, compactness, residue flexibility, and solvent exposure.

### 2.10. Western Blot Assay

Flies (40 per group) were homogenized in ice-cold RIPA lysis buffer supplemented with protease and phosphatase inhibitors (98:1:1, *v*/*v*/*v*), and lysed on ice for 15 min. After centrifugation at 14,000× *g* for 15 min at 4 °C, the supernatant was collected and total protein concentration was quantified using a BCA assay kit (CAS:1239-45-8, MCE, Shanghai China). Equal amounts of protein were mixed with SDS loading buffer and denatured at 95 °C for 10 min. Protein samples were separated by SDS-PAGE (80 V, 2 h) and transferred onto a methanol-activated PVDF membrane (200 mA, 90 min, ice bath). The membrane was blocked with rapid blocking solution (20 min, RT, shaking), followed by overnight incubation with primary antibody at 4 °C. After three 15 min washes with TBST, the membrane was incubated with HRP-conjugated secondary antibody (2 h, RT) and washed. Protein bands were visualized using an Omni-ECL chemiluminescence kit (Thermo Fisher, Wuhan, China) and imaged with a chemiluminescence imaging system. All primary and secondary antibodies used for Western blot analysis were purchased from Cell Signaling Technology (CST, Danvers, MA, USA), while other reagents for the Western blot assay were obtained from Wuhan Servicebio Technology Co., Ltd. (Wuhan, China).

### 2.11. Statistical Analysis

All statistical evaluations were performed using GraphPad Prism 9.5 (GraphPad Software, La Jolla, CA, USA). Results are expressed as mean ± standard deviation (SD). Comparisons between two groups were performed using an unpaired two-tailed Student’s *t*-test, and One-Way ANOVA for multiple group comparisons. Statistical significance levels are denoted as ns, no significance; *, *p* < 0.05; **, *p* < 0.01; ***, *p* < 0.001; ****, *p* < 0.0001. All data represent at least three independent biological replicates.

## 3. Results

### 3.1. XFBD Alleviates HSD-Induced T2DM-like Phenotypes in D. melanogaster

**Figure 1:**
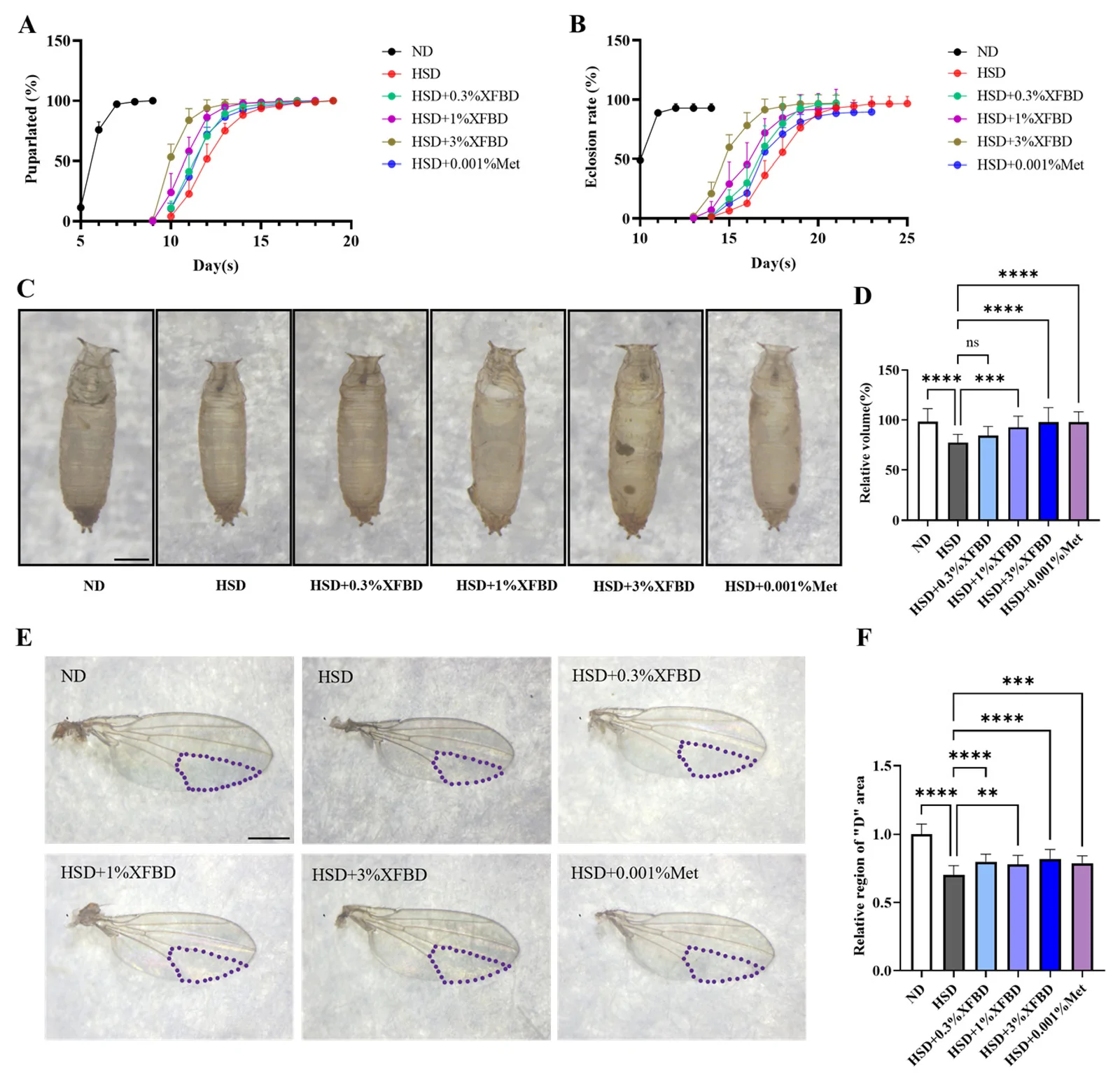
Effect of XFBD on the growth and development in an HSD-induced *D. melanogaster* larval T2DM model. (**A**) Pupation rate of flies fed ND, HSD, or HSD supplemented with 0.3%, 1%, or 3% XFBD, or 0.001% Met. (*n* = 5). (**B**) Eclosion rate (*n* = 5). (**C**) Representative images of pupae. Scale bar, 200 μm. (**D**) Quantification of relative pupal volume in each group (*n* = 20). (**E**) Representative images of adult fly wings, with the D area outlined by purple dashed lines. Scale bar, 500 μm. (**F**) Quantification of relative D area in wings of adult flies from each group (*n* = 20). Data were analyzed by One-Way ANOVA (ns, no significance; **, *p* < 0.01; ***, *p* < 0.001; ****, *p* < 0.0001).

XFBD composition was characterized by LC-Q-TOF-MS in our previous work, identifying 91 constituents, importantly, the XFBD used in this study and the XFBD used in the published research both come from the same batch. We first assessed the protective effect of XFBD against high-sugar diet-triggered developmental defects. HSD feeding is known to induce pathological phenotypes that resemble human MetS and T2DM in *Drosophila*, including developmental delay, hyperglycemia, and insulin resistance [52]. HSD significantly delayed pupariation and eclosion, and reduced pupal volume and adult wing size compared to ND-fed controls. XFBD treatment dose-dependently accelerated developmental progression: 3% XFBD shortened the time to 50% pupariation and eclosion by 3–4 days compared with the HSD group (Figure 1A,B). XFBD also significantly restored pupal volume at 1% and 3% concentrations, with effects comparable to those of the positive control metformin (Met, 0.001%) (Figure 1C,D). The wing D region area showed a restoring trend following XFBD treatment (Figure 1E,F). In summary, XFBD effectively ameliorates HSD-induced developmental toxicity.

**Figure 2:**
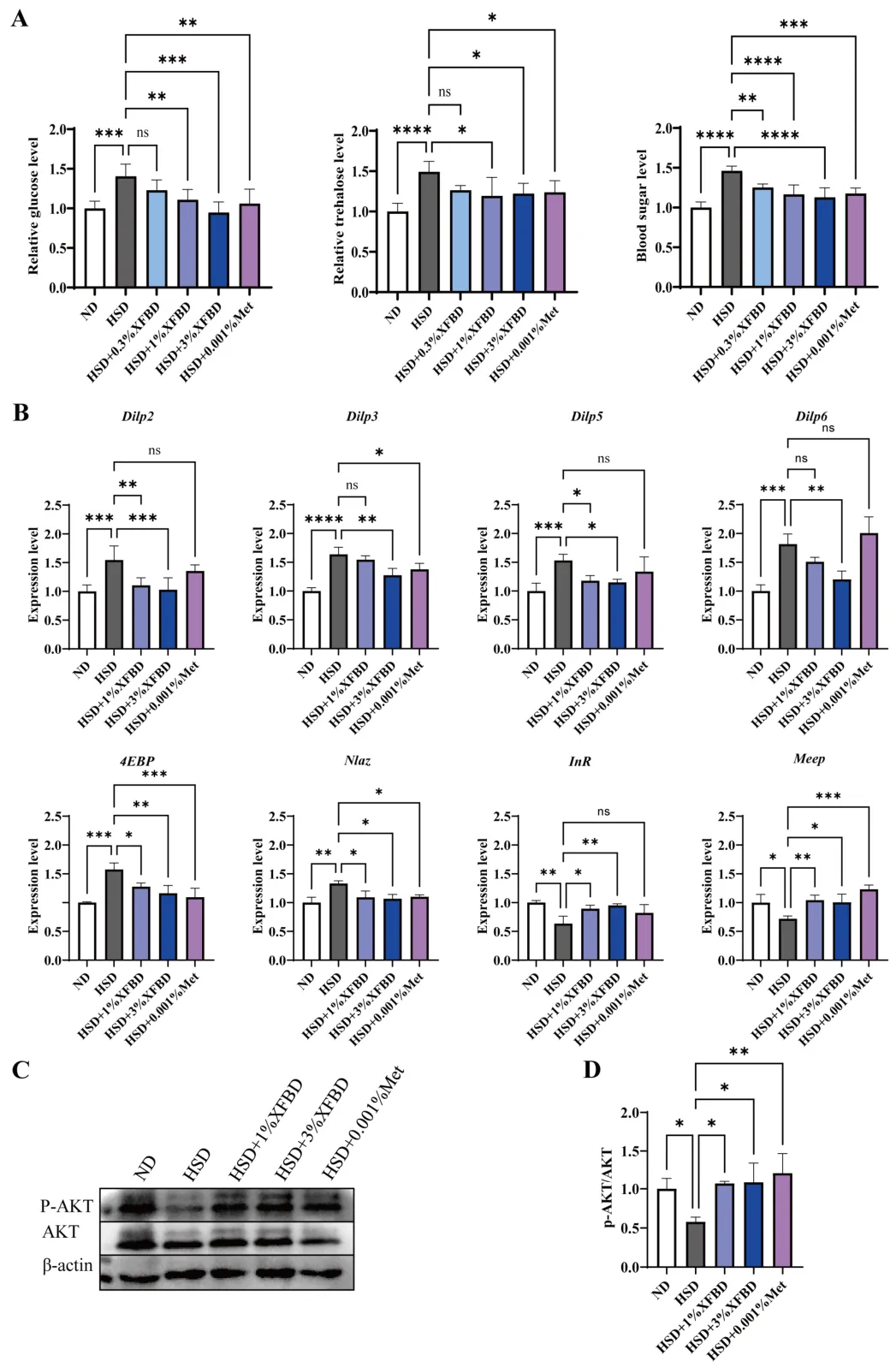
Effect of XFBD on HSD-induced hyperglycemia, and insulin resistance in *D. melanogaster* adults. (**A**) Relative glucose level, relative trehalose level, and total blood sugar level in flies of each group (*n* ≥ 3). (**B**) Relative mRNA expression levels of genes involved in the insulin signaling pathway in each group. (**C**) Western blot analysis of AKT phosphorylation levels. (**D**) Quantitative analysis of AKT phosphorylation levels. (*n* = 3) Data were analyzed by One-Way ANOVA (ns, no significance; *, *p* < 0.05; **, *p* < 0.01; ***, *p* < 0.001; ****, *p* < 0.0001).

In the adult HSD model (Figure 2), XFBD supplementation dose-dependently reduced hemolymph glucose, trehalose, and total sugar levels. At 3% XFBD, all sugar parameters approached ND levels, while 1% XFBD produced partial amelioration (Figure 2A). Based on these results, the 0.3% XFBD dose was excluded from subsequent adult-stage experiments. At the molecular level, HSD significantly elevated mRNA expression of insulin-like peptides (*dilp2*, *dilp3*, *dilp5*, *dilp6*) and the insulin resistance markers *4EBP* and *Nlaz* compared with ND controls, along with significantly downregulated expression of *InR* and *Meep* [53,54,55]. XFBD treatment reversed the aberrant expression of these genes toward ND levels in a dose-dependent manner (Figure 2B). Importantly, HSD significantly reduced AKT phosphorylation at Ser-473, a key functional readout of insulin signal transduction, and XFBD treatment (3%) significantly restored p-AKT levels (Figure 2C,D). These results indicate that HSD triggers systemic insulin resistance, and XFBD dose-dependently improves insulin signaling to alleviate HSD-induced insulin resistance. Metformin showed partial rescue effects on *dilp3*, *4EBP*, *Nlaz*, and p-AKT, whereas XFBD exhibited broader and more robust efficacy.

Collectively, these findings indicate that XFBD alleviates HSD-induced developmental impairment and restores glycemic control and insulin signaling—two hallmark features of T2DM with direct therapeutic relevance.

### 3.2. XFBD Mitigates HSD-Induced Glycogen and Lipid Metabolism Disorders

**Figure 3:**
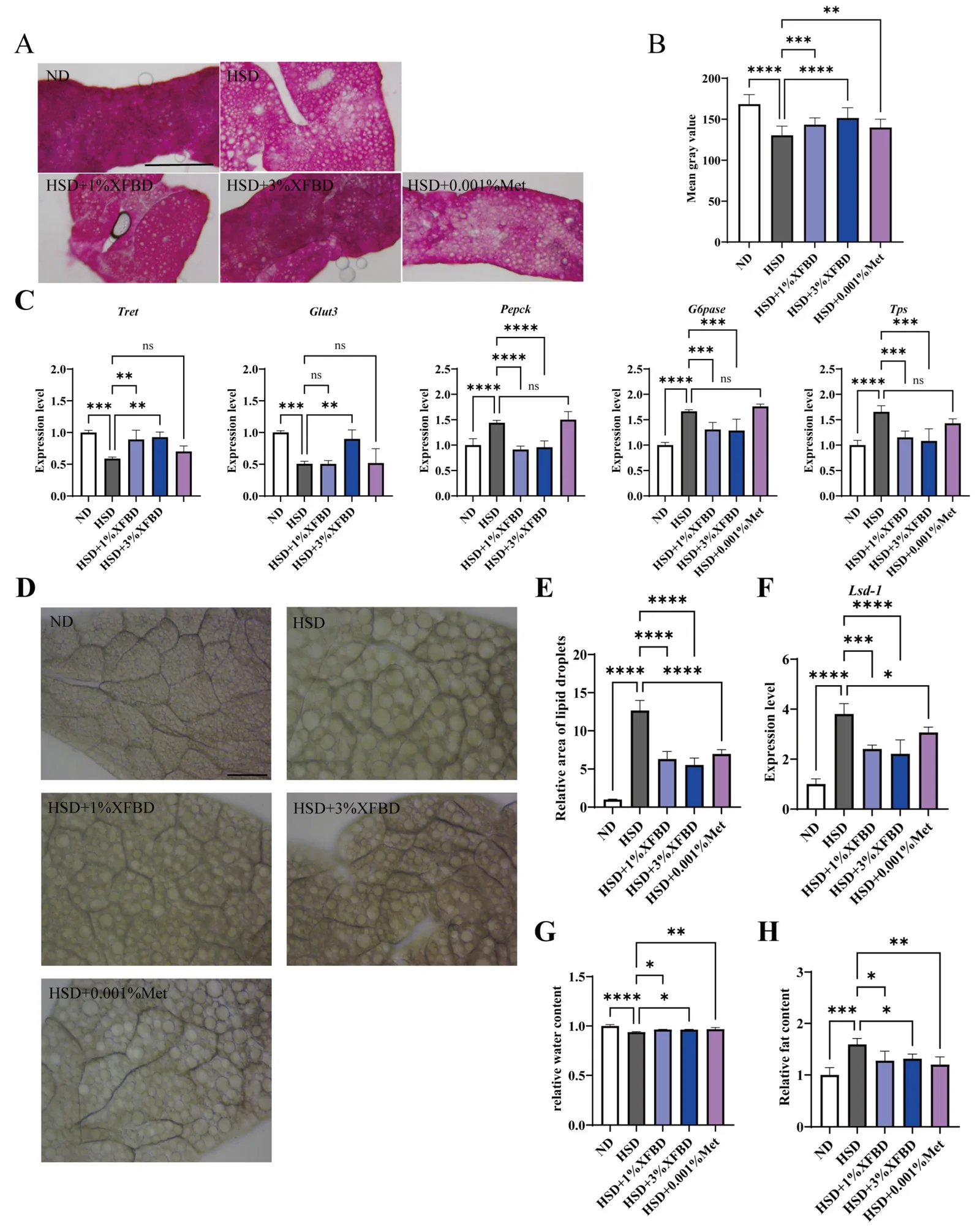
Effect of XFBD on HSD-induced glycogen metabolism disorders and lipid accumulation. (**A**) PAS staining of glycogen in the fat body. Scale bar: 200 μm. (**B**) Quantitative analysis of the mean gray value of glycogen staining. (*n* = 20). (**C**) Relative mRNA expression levels of glucose metabolism-related genes. (**D**) Representative images of lipid droplets in the fat body of each group. Scale bar: 50 μm. (**E**) Quantitative analysis of the relative area of lipid droplets. (**F**) Relative mRNA expression level of lipid metabolism-related genes. (**G**) Relative water content. (**H**) Relative fat content. Data were analyzed by One-Way ANOVA (ns, no significance; *, *p* < 0.05; **, *p* < 0.01; ***, *p* < 0.001; ****, *p* < 0.0001).

Glycogen serves as a core energy reserve, and its metabolic balance is crucial for maintaining energy homeostasis [56]. HSD significantly reduced glycogen content in the fat body as revealed by PAS staining, and XFBD treatment (1% and 3%) significantly restored glycogen storage (Figure 3A,B). Trehalose, a major circulating sugar in *D. melanogaster*, also contributes to energy homeostasis [57]. XFBD normalized the expression of trehalose metabolism genes (*Tps*, *Tret*) and reversed HSD-induced dysregulation of the glucose transporter gene (*Glut3*) and gluconeogenesis genes (*Pepck*, *G6Pase*) (Figure 3C).

HSD also upregulates lipogenesis, subsequently leading to lipid accumulation [58]. HSD induced significant lipid droplet enlargement and upregulated *lipid storage droplet 1* (*Lsd-1*) expression in the fat body (Figure 3D–F). *Lsd-1* is the *Drosophila* ortholog gene of mammalian *perilin-1*, a lipid droplet surface gene governing triglyceride lipolysis and systemic lipid homeostasis under high-sugar metabolic stress [59]. Both 1% and 3% XFBD significantly reduced lipid droplet size and *Lsd-1* gene expression to near-ND levels (Figure 3D–F). Body weight and water/lipid content analysis confirmed reduced water content and elevated lipid content in HSD-fed flies, both significantly restored by XFBD treatment (Figure 3G,H). Metformin showed comparable effects on lipid parameters.

Collectively, these findings suggest that XFBD ameliorates HSD-triggered carbohydrate and lipid metabolic dysfunction in the *Drosophila* fat body through recovering glycogen and trehalose reserves and attenuating excessive lipogenic deposition.

### 3.3. Metabolomics Reveals XFBD Reverses T2DM-Associated Metabolic Reprogramming

**Figure 4:**
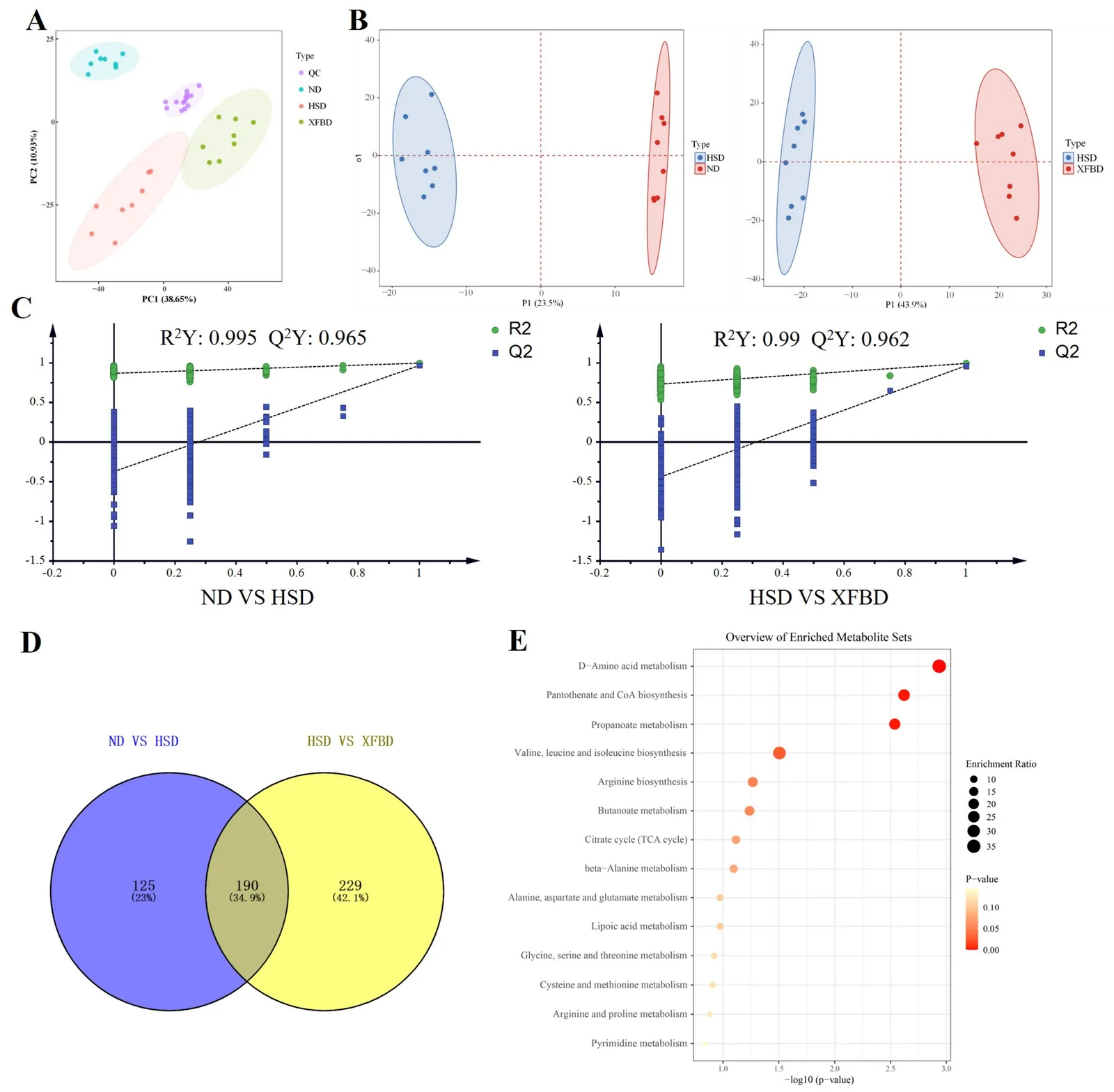
Effect of XFBD on HSD-induced untargeted metabolomics in *D. melanogaster*. (**A**) PCA score plot showing the separation of experimental samples (ND, HSD and XFBD groups) and quality control (QC) samples. (**B**) OPLS-DA score plots for ND vs. HSD and HSD vs. XFBD comparisons. (**C**) Permutation test plots of OPLS-DA models for ND vs. HSD and HSD vs. XFBD. (**D**) Venn diagram of differential metabolites between ND vs. HSD and HSD vs. XFBD. (**E**) KEGG pathway enrichment bubble plot of differential metabolites. Dot size represents enrichment ratio, and the color gradient indicates *p*-value.

To further evaluate the intervention effect of XFBD on metabolic disorders in a *Drosophila* T2DM model, non-targeted metabolomics was conducted across three groups: the ND, HSD, and 3% XFBD. Principal component analysis (PCA) showed distinct clustering and separation of samples from different groups along the principal component axes, indicating significant differences in metabolite profiles under different treatments (Figure 4A). Tight clustering of quality control (QC) samples confirmed the high quality and reliability of the data. Orthogonal partial least squares discriminant analysis (OPLS-DA) score plots demonstrated complete separation between ND vs. HSD and HSD vs. XFBD (Figure 4B). The stability and predictive power of the models were validated using permutation tests. The coefficient of determination (R^2^Y) and predictive ability (Q^2^Y) of both models were close to 1 (ND vs. HSD: R^2^Y = 0.995, Q^2^Y = 0.965; HSD vs. XFBD: R^2^Y = 0.99, Q^2^Y = 0.962), and the intercepts of R^2^ and Q^2^ in permutation tests satisfied the criteria for model reliability (Figure 4C), confirming that the OPLS-DA models were stable and exhibited excellent predictive performance.

Using variable importance in projection (VIP) > 1.0 and *p* < 0.05 as screening thresholds, a total of 315 differential metabolites were screened in the comparison between normal diet (ND) and high-sugar diet (HSD), and 419 metabolites were screened in the comparison between HSD and XFBD. Notably, 190 metabolites were found to overlap between these two comparisons (Figure 4D). Further refinement using fold change (FC) criteria, specifically FC > 1.2 (upregulation) and FC < 0.8 (downregulation), resulted in the identification of 79 metabolites that were aberrantly expressed in the HSD group compared with the ND group, which were significantly reversed by the XFBD intervention (Table S2). These metabolites are proposed as candidate biomarkers that underlie the anti-diabetic effects of XFBD. To elucidate their biological functions, KEGG pathway enrichment analysis was conducted utilizing MetaboAnalyst. The results indicated significant enrichment in multiple core metabolic pathways closely associated with the pathogenesis of T2DM, including D-amino acid metabolism, pantothenate and coenzyme A (CoA) biosynthesis, valine/leucine/isoleucine biosynthesis, arginine biosynthesis, and the citrate cycle (TCA cycle) (Figure 4E). The dysregulation of these pathways is a prominent feature of T2DM development and progression, involving key pathological processes such as insulin resistance, mitochondrial dysfunction, and chronic inflammation [60,61,62,63].

### 3.4. XFBD Does Not Exert Its Effects via Modulation of the Gut Microbiota

**Figure 5:**
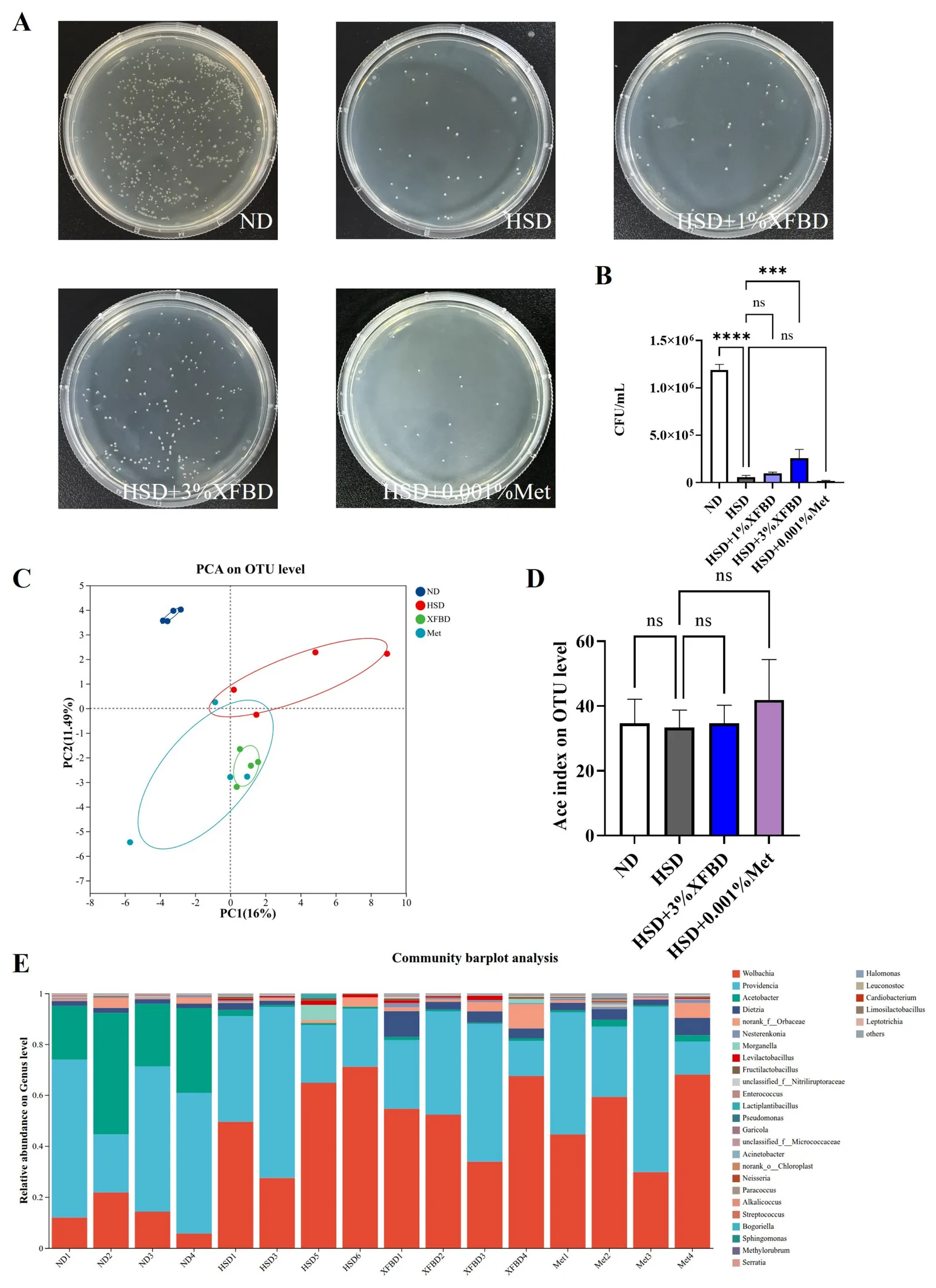
XFBD exerts no significant regulatory effect on HSD-induced gut microbiota dysbiosis in *D. melanogaster*. (**A**) Representative images of LB plate cultures of gut microbiota in each group. (**B**) Statistical results of CFU/mL of gut microbiota. (**C**) PCA of gut microbiota. (**D**) Ace index analysis of gut microbiota at the OTU level. (**E**) Stacked bar plot of gut microbiota community composition at the genus level. Data were analyzed by One-Way ANOVA (ns, no significance; ***, *p* < 0.001; ****, *p* < 0.0001).

To assess whether gut microbiota modulation contributes to the anti-T2DM effects of XFBD, we first measured the colony-forming units (CFU) in each group. CFU were significantly reduced in HSD-fed flies relative to ND controls, indicating that HSD stress markedly decreased gut microbial community abundance, consistent with disruption of gut microecological homeostasis under HSD conditions. Treatment with 3% XFBD produced a slight increasing trend in CFU, but counts remained far below ND levels; 1% XFBD and metformin showed no apparent recovery (Figure 5A,B).

To further dissect structural changes in the gut microbiota, 16S rRNA high-throughput sequencing was performed. Four experimental groups were established: ND, HSD, XFBD (3%), and Met (0.001%). PCA based on OTU levels revealed a clear separation between the ND and HSD groups, confirming that HSD significantly reshaped the overall gut microbiota structure. However, samples from the XFBD and Met groups clustered closely with those of the HSD group without obvious separation, indicating that XFBD intervention failed to reverse the HSD-induced restructuring of the gut microbiota (Figure 5C). α-diversity analysis further demonstrated no significant differences in the Ace index among the ND, HSD, and XFBD/Met treatment groups (Figure 5D).

Community composition analysis at the genus level (Figure 5E) showed that the beneficial bacterium Acetobacter was significantly reduced in the HSD group compared with the ND group, with no obvious reversal in the XFBD group. Wolbachia abundance was markedly elevated in the HSD group, and neither XFBD nor Met effectively reduced its abundance. Meanwhile, no significant differences in the relative abundance of other genera were observed among groups, further supporting that XFBD did not produce substantial regulatory effects on gut microbiota composition. Since 16S rRNA sequencing only reflects relative taxonomic proportions rather than absolute bacterial counts, we further performed colony counting to directly quantify absolute loads of the two dominant commensal symbionts *Acetobacter* and *Lactobacillus*. Consistent with the sequencing data, HSD exposure sharply reduced the colony-forming units (CFU) of both strains. Neither 3% XFBD nor 0.001% metformin could reverse this depletion (Figure S1).

Collectively, these findings consistently demonstrate that XFBD does not exert a substantial modulatory effect on gut microbiota composition in this HSD-induced T2DM model. Its anti-diabetic effects are not mediated through modulation of the gut microbiome.

### 3.5. XFBD Suppresses Aberrant JNK Pathway Activation

**Figure 6:**
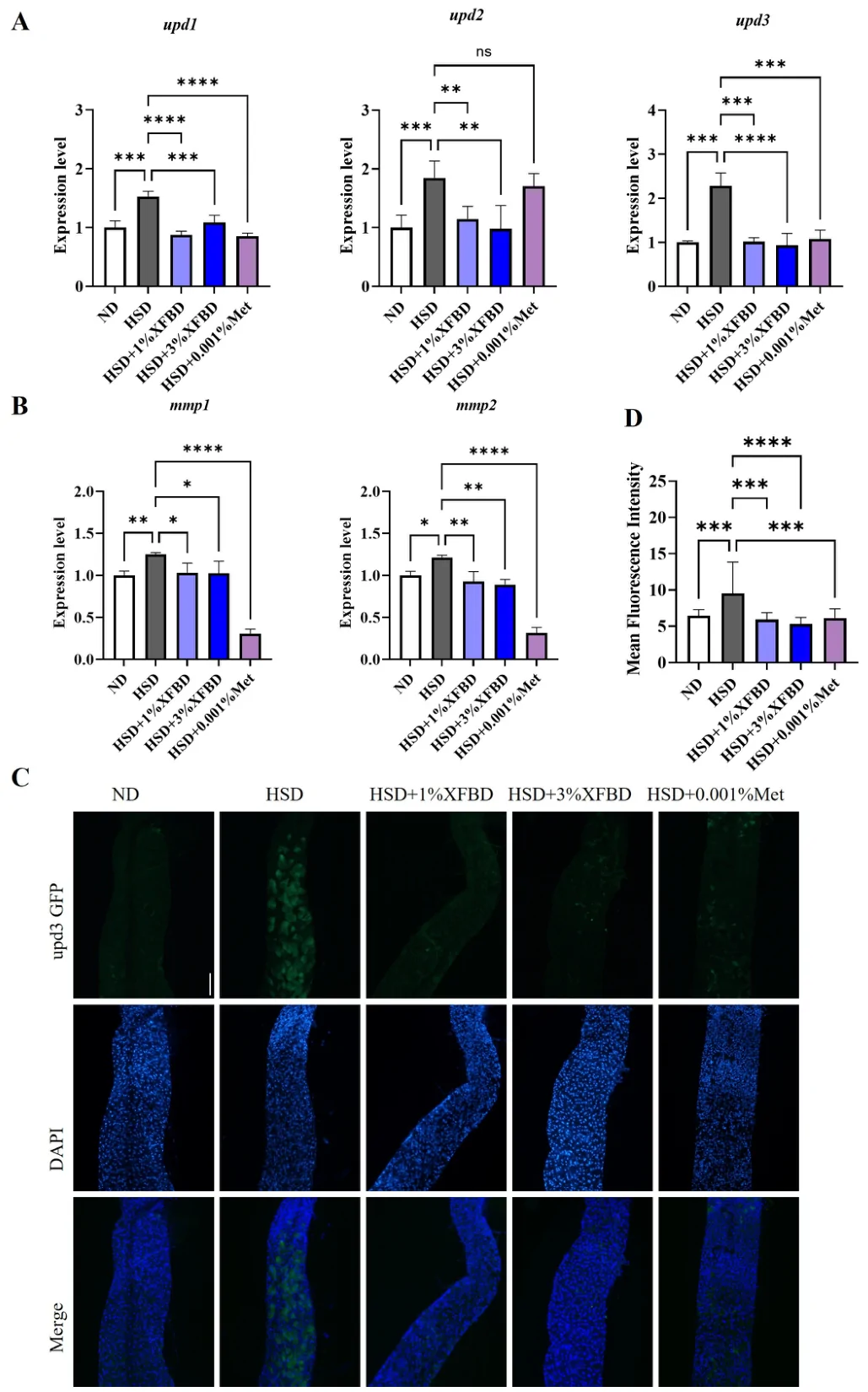
Effect of XFBD on the expression of the JNK signaling pathway in the HSD-induced *D. melanogaster* T2DM model. (**A**) Relative mRNA expression levels of JNK downstream target genes—including the Upd family cytokines (*upd1*, *upd2*, *upd3*). (**B**) Relative mRNA expression levels of JNK downstream target genes—matrix metalloproteinases (*mmp1*, *mmp2*). (**C**) Representative *upd3*-GFP immunofluorescence images. *upd3* GFP (green) labels *upd3* expression, DAPI (blue) labels nuclei, and Merge shows the merged images. Scale bar, 50 μm. (**D**) Quantitative analysis of upd3 GFP fluorescence intensity. Data were analyzed by One-Way ANOVA (ns, no significance; *, *p* < 0.05; **, *p* < 0.01; ***, *p* < 0.001; ****, *p* < 0.0001).

JNK cascade hyperactivation is a central driver of high-calorie diet-triggered insulin resistance, and its suppression alleviates hyperglycemia and insulin resistance in *Drosophila* models [15,64]. Compared with the ND group, mRNA expression of JNK downstream target genes—including the Upd family cytokines (*upd1*, *upd2*, *upd3*) (Figure 6A) and matrix metalloproteinases (*mmp1*, *mmp2*) (Figure 6B)—was significantly upregulated in the HSD group. XFBD or metformin treatment significantly reversed this upregulation, restoring expression toward ND baselines (Figure 6A,B). We further utilized *upd3*-Gal4 > UAS-GFP transgenic *D. melanogaster* to visualize upd3 expression (Figure 6C,D). Consistent with the gene expression trends, HSD-induced GFP upregulation in upd3-Gal4 > UAS-GFP flies were reversed by XFBD or metformin. These findings establish that XFBD negatively regulates JNK pathway activity, counteracting HSD-driven pathway hyperactivation, thereby suppressing inflammatory cytokine expression (e.g., Upd3) and ameliorating insulin signaling impairment. Consistently, XFBD also effectively attenuated HSD-induced oxidative stress—a downstream consequence of hyperglycemia and JNK pathway activation—as evidenced by reduced intestinal ROS levels and restored expression of antioxidant defense genes (*cat*, *Sod1*, *Sod2*, *cnc*) (Figure S2). To confirm that JNK activity is required for HSD-induced hyperglycemia, we expressed a dominant-negative form of *Drosophila* JNK (*bsk*^DN^) under HSD feeding. *bsk*^DN^ largely reversed the HSD-induced elevation of hemolymph glucose and total sugar, showing a concordant downward trend (Figure S5D–F). This result directly demonstrates that JNK activity is necessary for the full hyperglycemic phenotype of HSD, and inhibition of JNK pathway hyperactivity effectively alleviates HSD-induced insulin resistance.

### 3.6. XFBD Inhibits the JAK/STAT Signaling Pathway

**Figure 7:**
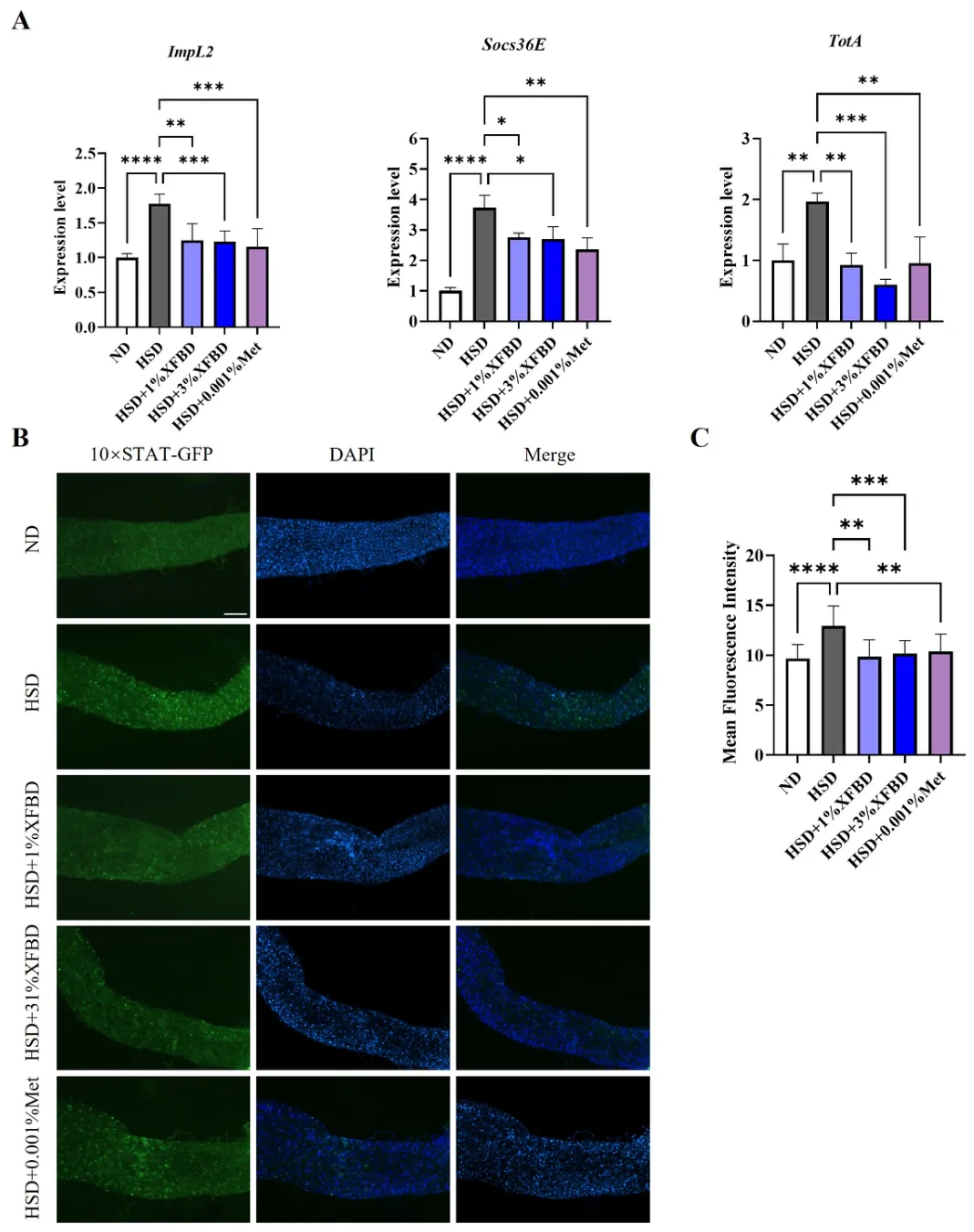
Effect of XFBD on the JAK/STAT signaling pathway in the HSD-induced *D. melanogaster* T2DM model. (**A**) Relative mRNA expression levels of JAK/STAT pathway target genes detected by RT-qPCR. (**B**) Representative immunofluorescence images of the 10 × STAT GFP reporter (green) and DAPI (blue) in *D. melanogaster* midgut tissues across groups. Merged images are shown on the right. Scale bar, 50 μm. (**C**) Quantitative analysis of 10 × STAT-GFP fluorescence intensity. Data were analyzed by One-Way ANOVA (*, *p* < 0.05; **, *p* < 0.01; ***, *p* < 0.001; ****, *p* < 0.0001).

The JAK/STAT signaling pathway is a central mediator of inflammatory responses and a critical contributor to insulin resistance [17,47,65]. Targeted suppression of JAK/STAT signaling rescues HSD-induced insulin resistance in *Drosophila* [66,67]. RT-qPCR was performed to detect the expression levels of key downstream genes of the JAK/STAT pathway (Figure 7A). mRNA expression of *ImpL2*, *Suppressor of cytokine signaling at 36E* (*Socs36E*), and *Turandot A* (*TotA*) was significantly upregulated in the HSD group, and downregulated by XFBD or metformin to levels comparable to ND. The 10 × STAT-GFP transgenic *Drosophila* was employed to monitor JAK/STAT pathway activity in vivo. The GFP fluorescence intensity in the intestinal tissues of the HSD group was significantly higher than that in ND, indicating that HSD induces JAK/STAT pathway activation. XFBD or metformin intervention markedly attenuated this increase (Figure 7B,C). In conclusion, XFBD inhibits JAK/STAT hyperactivation, which may represent an additional key mechanism underlying its anti-diabetic effects. To establish the causal role of JAK/STAT signaling in HSD-induced hyperglycemia, we performed complementary gain- and loss-of-function experiments. Ubiquitous overexpression of *upd3*—the key cytokine ligand that activates the *Drosophila* JAK/STAT pathway—was sufficient to significantly elevate hemolymph glucose, trehalose, and total sugar levels under normal-diet conditions (Figure S5A–C), demonstrating that JAK/STAT pathway activation is sufficient to perturb systemic sugar homeostasis. Pharmacological inhibition of JAK/STAT signaling with methotrexate (MTX), a compound reported to inhibit JAK1/JAK2 and downstream STAT activation [68], significantly attenuated HSD-induced elevation of hemolymph glucose and total sugar, with trehalose showing a concordant downward trend (Figure S5G–I). Together, these gain- and loss-of-function results establish that JAK/STAT signaling is both sufficient to drive hyperglycemia and required for the full HSD phenotype, supporting the conclusion that XFBD ameliorates insulin resistance, at least in part, through suppression of this axis.

### 3.7. Network Pharmacology and Computational Analyses Identify Glycyrrhizic Acid (GA) as a Core Active Component

**Figure 8:**
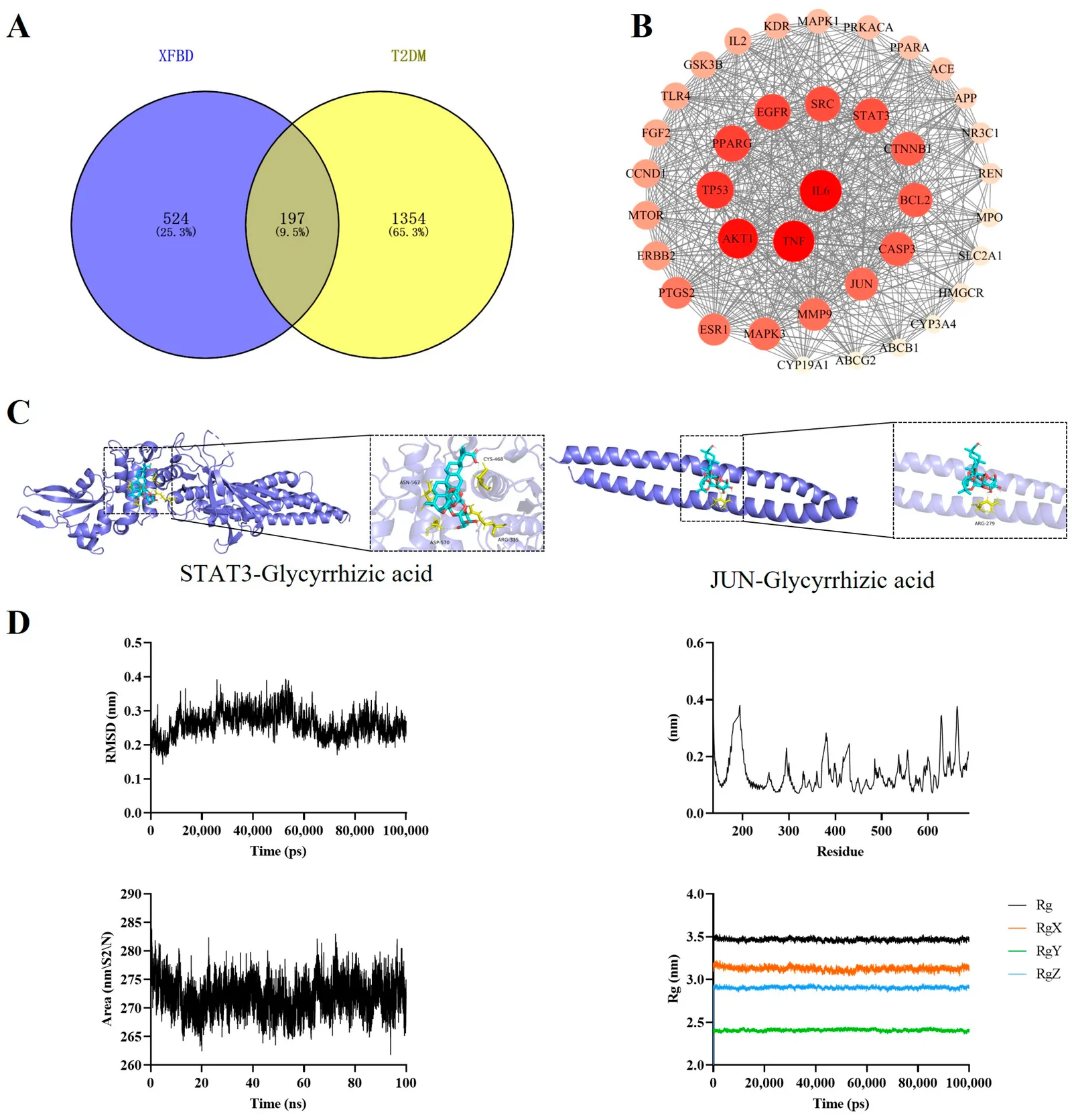
Network pharmacology and computational analyses identify core active components and core targets. (**A**) Venn diagram of XFBD targets and T2DM targets. (**B**) PPI network of 38 potential core targets of XFBD in the treatment of T2DM. (**C**) Molecular docking diagrams of STAT3 and JUN with GA. (**D**) Molecular dynamics simulation of the STAT3-GA complex.

To identify the pharmacologically active constituents responsible for XFBD’s anti-T2DM effects, network pharmacology was applied. The 91 chemical constituents that have been identified and reported in the XFBD extract [32] were subjected to the SwissTarget-Prediction tool, yielding 721 potential targets, with 197 overlapping T2DM-associated targets from GeneCards, OMIM, and TTD databases (Figure 8A and Figure S3A). PPI network analysis of the 197 overlapping targets (Figure S3B), constructed in STRING and analyzed in Cytoscape, identified a core subnetwork of 38 hub targets (Figure 8B). This core network included JUN (a central component of the JNK signaling pathway) and STAT3 (the key transcriptional effector of the JAK/STAT pathway), directly corroborating our experimental findings. KEGG enrichment analyses of the 197 targets highlighted insulin resistance, PI3K-Akt signaling, and AGE-RAGE signaling in diabetic complications as top enriched pathways (Figure S3C), further supporting the relevance of the predicted target to T2DM pathophysiology.

Based on the above analysis, molecular docking of STAT3 (PDB ID: 6NJS) and JUN (PDB ID: 1JNM) with the corresponding active components identified in XFBD was performed using AutoDock Vina. Glycyrrhizic acid (GA), a triterpenoid saponin abundant in *Glycyrrhizae Radix et Rhizoma*, exhibited the most favorable binding energies with both STAT3 (−9.5 kcal/mol) and JUN (−7.8 kcal/mol) (Table 1), surpassing other candidate compounds including uralsaponin C (−9.0 and −7.2 kcal/mol, respectively), glabrolide (−7.1 kcal/mol), and atractylenolide I (−6.6 kcal/mol) (Figure S3D, Table 1).

To further validate the molecular docking results, molecular dynamics simulations were performed for GA, the active component exhibiting the lowest binding energies to both core targets, STAT3 and JUN (Figure 8D). Key parameters including root mean square deviation (RMSD), root mean square fluctuation (RMSF), radius of gyration (Rg), and solvent accessible surface area (SASA) were analyzed to evaluate the binding stability and conformational changes in of the receptor-ligand complexes. The simulation results demonstrated that the GA-STAT3 complex exhibited stable behavior across all parameters, indicating high binding affinity and a compact conformation. In contrast, the GA-JUN complex showed relatively greater conformational fluctuations (Figure S3E), suggesting weaker binding stability and more dynamic binding, which suggests that GA may form a more stable complex with STAT3 than with JUN, whereas the effects of GA on the JUN pathway may not be mediated through direct binding.

### 3.8. GA Recapitulates XFBD’s Hypoglycemic and Dual Pathway-Inhibitory Effects In Vivo

**Figure 9:**
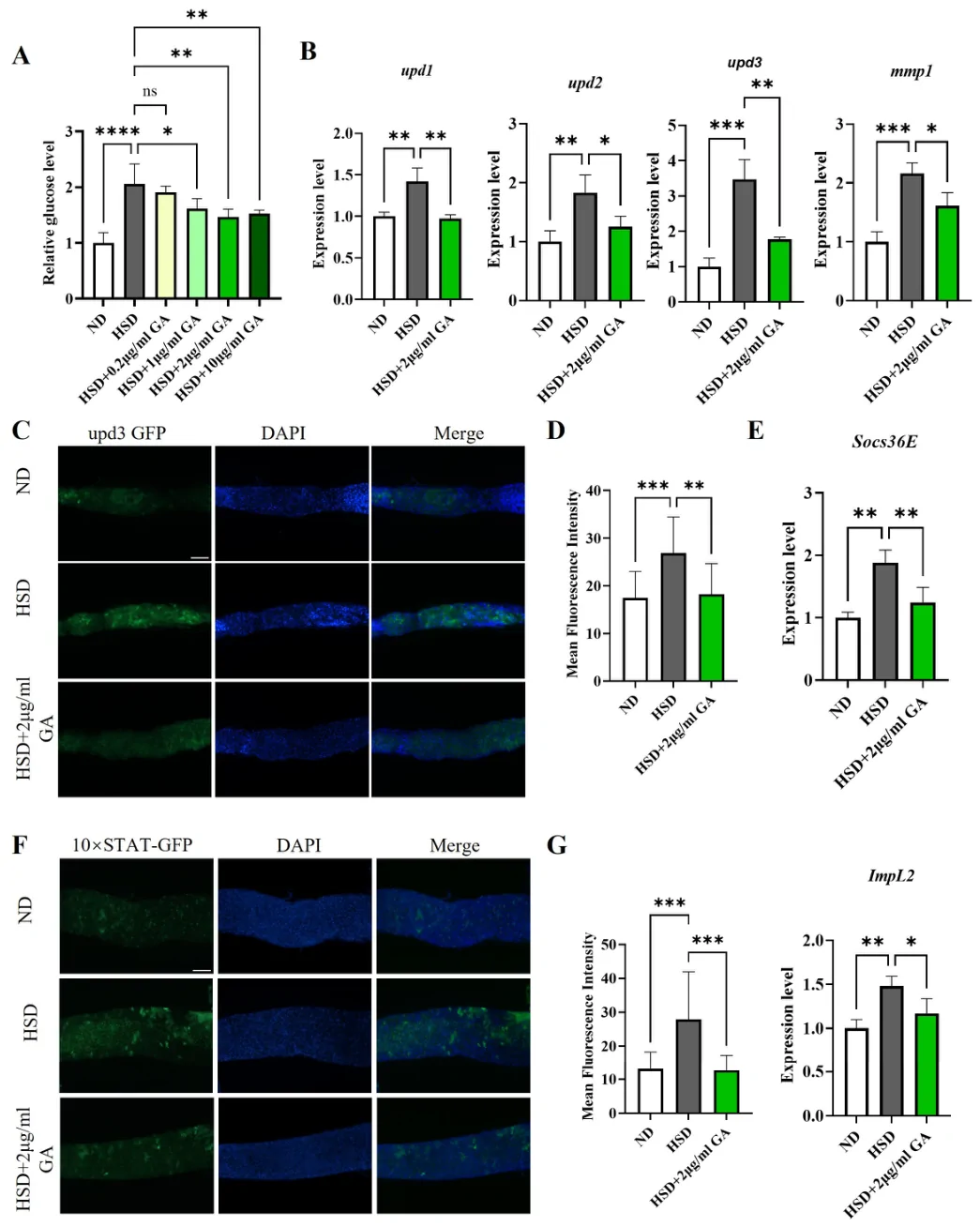
Effect of GA on hyperglycemia in HSD-induced *D. melanogaster* T2DM model. (**A**) Relative glucose level in flies of each group (*n* ≥ 3). (**B**) Relative mRNA expression levels of JNK pathway target genes detected by RT-qPCR. (**C**) Representative upd3-GFP immunofluorescence images. upd3-GFP (green), DAPI (blue), and Merge shows the merged images. Scale bar, 50 μm. (**D**) Quantitative analysis of upd3 GFP fluorescence intensity. (**E**) Relative mRNA expression levels of JAK/STAT pathway target genes detected by RT qPCR. (**F**) Representative immunofluorescence images of the 10 × STAT-GFP reporter (green) and DAPI (blue) in *D. melanogaster* midgut tissues across groups. Merged images are shown on the right. Scale bar, 50 μm. (**G**) Quantitative analysis of 10 × STAT-GFP fluorescence intensity. Data were analyzed by One-Way ANOVA (ns, no significance; *, *p* < 0.05; **, *p* < 0.01; ***, *p* < 0.001; ****, *p* < 0.0001).

To experimentally validate the computational predictions, we tested GA across a four-concentration gradient (0.2, 1, 2, and 10 μg/mL) in the HSD-induced *Drosophila* T2DM model. GA produced a dose-dependent reduction in hemolymph glucose levels (Figure 9A). Low concentrations of GA (0.2 μg/mL and 1 μg/mL) did not significantly reduce glucose or total sugar levels, whereas 2 and 10 μg/mL GA significantly lowered both glucose and total sugar. Furthermore, the 2 μg/mL GA treatment demonstrated a stronger glucose-lowering effect (Figure 9A and Figure S4); therefore, 2 μg/mL was selected for subsequent mechanistic analyses.

At the mechanistic level, GA (2 μg/mL) recapitulated the dual pathway inhibitory effects observed with the full XFBD formula. GA significantly downregulated JNK target genes *upd1*, *upd2*, *upd3* and *mmp1* (*p* < 0.05–0.01 vs. HSD) (Figure 9B) and markedly reduced *upd3*-GFP fluorescence intensity in the *Drosophila* midgut (*p* < 0.01 vs. HSD) (Figure 9C,D). Concurrently, GA downregulated JAK/STAT target genes *ImpL2* and *Socs36E* (*p* < 0.05–0.01 vs. HSD) (Figure 9E) and significantly attenuated 10 × STAT-GFP reporter signal (*p* < 0.001 vs. HSD) (Figure 9F,G).

Collectively, 2 μg/mL GA produced effects comparable in both magnitude and pattern to 3% XFBD, supporting a key role for GA in mediating XFBD’s anti-T2DM effects through JNK/JAK-STAT pathway inhibition.

## 4. Discussion

XFBD is a well-characterized traditional Chinese medicine formula with robust anti-inflammatory and immunomodulatory activities, and has demonstrated clinical benefits in COVID-19 [33,69]. Mechanistically, its protective effects in these inflammatory disorders are consistently attributed to two core pharmacological properties: suppression of multiple pro-inflammatory cascades including NF-κB, JNK, and JAK/STAT signaling, and restoration of gut microbiota homeostasis [70,71]. Our previous study has demonstrated that XFBD alleviates intestinal inflammation and modulates microbiota distribution in a *Drosophila* IBD model by suppressing IMD/NF-κB and JNK signaling pathways, providing the first proof-of-concept that XFBD possesses efficacy against chronic inflammatory conditions of the gut [34]. This finding has since been corroborated in a mouse model of DSS-induced colitis, where XFBD ameliorated intestinal injury through the same NF-κB–dependent mechanism, alongside restoration of T-cell immunity and gut microbiota homeostasis [72]. Similarly, XFBD has been shown to protect against sepsis-induced acute lung injury by modulating gut microbial homeostasis and promoting inflammation resolution [73]. These evidences indicate that the mechanism by which XFBD suppresses inflammation through the NF-κB, JNK, and JAK/STAT pathways is conserved from insects to mammals.

Gut microbiota modulation was initially proposed as a candidate mechanism underlying XFBD’s metabolic benefits, given its well-documented microbiota-regulating effects in inflammatory disease models [34]. HSD induces significant gut microbial community shifts in *Drosophila*, which drive intestinal JNK-JAK/STAT activation and systemic insulin resistance, and supplementation with beneficial bacteria (*Acetobacter* and *Lactobacillus*) alleviates these metabolic phenotypes [74]. However, our combined 16S rRNA sequencing and culture-based CFU analysis consistently showed that XFBD failed to rescue HSD-induced microbial load reduction, reverse community compositional restructuring, or restore the abundance of key beneficial genera such as *Acetobacter* in the *Drosophila* gut (Figure 5). This negative finding may be explained by the distinct nature of dysbiosis between the two disease models, and it indicates that, at least in the *Drosophila* T2DM model, XFBD exerts anti-T2DM effects through microbiota-independent, host-directed mechanisms, distinguishing its mode of action from that of prebiotic-dependent anti-diabetic natural products [75].

**Figure 10:**
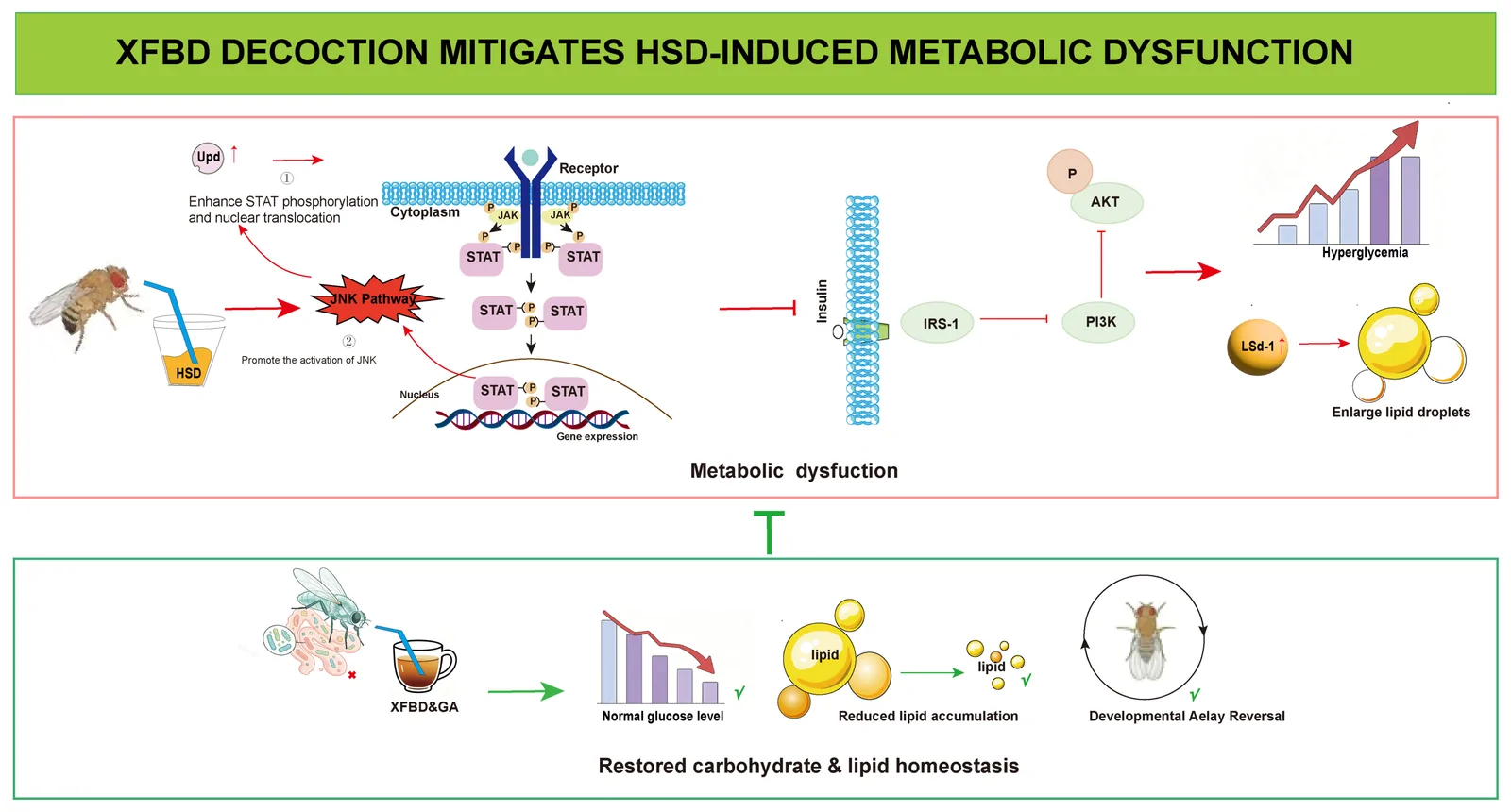
Mechanism diagram of XFBD improving HSD-triggered metabolic disorders in *Drosophila.* HSD activates the JNK pathway in *Drosophila*. Stimulated JNK signaling induces transcription of the IL-6-like cytokine Upd3, which subsequently activates the downstream JAK/STAT pathway, constructing a harmful JNK/JAK/STAT inflammatory axis. Continuous hyperactivation of this axis drives lipid droplet expansion, hyperglycemia and metabolic dysfunction. XFBD and its main active constituent GA suppress the overactivated JNK/JAK/STAT inflammatory cascade. Blockade of this inflammatory axis rescues defective IRS-1/PI3K/Akt insulin signaling, relieves lipid deposition and hyperglycemia, and restores normal carbohydrate and lipid metabolism. Red arrows denote activation or upregulation; green arrows denote beneficial effects; T-bars denote inhibition.

Instead, our data demonstrate that simultaneous suppression of the JNK and JAK/STAT inflammatory axes accounts for the broad metabolic benefits of XFBD (Figure 6 and Figure 7). These two pathways do not operate independently in metabolic disease; rather, they form a self-amplifying positive feedback loop that acts as a core pathological hub not only for insulin resistance, but also for other cardinal features of metabolic syndrome including lipotoxicity, oxidative stress and systemic metabolic dysregulation [20,21]. Our previous research characterized the conserved pathway architecture in *Drosophila*: HSD-induced gut microbiota dysbiosis triggers intestinal JNK pathway activation, which upregulates upd cytokines (the *Drosophila* functional homologs of mammalian IL-6) that in turn activate JAK/STAT signaling—creating a self-amplifying feed-forward inflammatory loop that drives systemic insulin resistance, a mechanism highly conserved with mammalian obesity-associated IR [74]. These pathway-inhibitory effects of XFBD are consistent with prior independent observations in mouse pneumonia and acute lung injury models [71,76], in *Drosophila* IBD models [34], as well as from network pharmacology-based predictions [34]. Our findings expand the disease scope of XFBD by validating its anti-diabetic efficacy via inhibition of the JNK/JAK-STAT inflammatory axis (Figure 10). Nevertheless, it should be emphasized that the JNK/JAK-STAT axis identified here likely represents one of several mechanisms through which XFBD improves insulin sensitivity, rather than an exclusive pathway. As a multi-herb formula, XFBD may also act on additional targets—such as other inflammatory cascades, metabolic enzymes, or gut microbiota–host signaling axes—that remain to be dissected in future studies.

The untargeted metabolomics data further support a systemic metabolic regulatory effect of XFBD that extends beyond glucose-lowering alone (Figure 4). D-amino acids are a special class of intercellular signaling molecules that have been detected in rodent and human pancreatic islets, among which D-alanine and D-serine are closely related to the regulation of insulin secretion [77]. Lee et al. found that the percentage of D-serine in pancreatic islets of T2DM patients was significantly reduced, and both D-serine and D-aspartate levels were negatively correlated with Glycosylated Hemoglobin A1c (HbA1c), suggesting that D-amino acid metabolic disturbances can directly impair pancreatic β-cell function and thereby contribute to T2DM progression [78]. Pantothenate is a key precursor for CoA synthesis, and CoA serves as an essential cofactor in carbohydrate, lipid, and protein metabolism [79,80], playing an indispensable role in the TCA cycle, fatty acid oxidation, and amino acid metabolism. In the HSD-induced *Drosophila* T2DM model, dietary supplementation with pantothenate effectively ameliorated HSD-induced free fatty acid accumulation and hyperglycemia, indicating that the pantothenate/CoA pathway exerts an important metabolic protective role under high-glucose stress, and that dysfunction of this pathway is closely associated with the onset and progression of T2DM [81]. Branched-chain amino acids (including valine, leucine, and isoleucine) and arginine are essential amino acids. Evidence indicates that BCAAs and arginine can exacerbate insulin resistance by interfering with insulin receptor signaling [82,83,84]. The TCA cycle is a central pathway of energy metabolism and the common terminal pathway for the oxidation of carbohydrates, fats, and amino acids [85,86,87]. Disturbances in TCA cycle-related metabolites can directly lead to insufficient mitochondrial energy supply, thereby inducing and aggravating insulin resistance [88]. Collectively, these pathways span glucose homeostasis, amino acid metabolism and energy production, representing the core metabolic dysregulations underlying T2DM. In line with the restored AKT phosphorylation and suppressed JNK/JAK-STAT activity, the metabolomic recovery induced by XFBD and GA likely reflects resolution of insulin resistance rather than direct targeting of metabolic enzymes. Given that impaired insulin signaling is known to disrupt BCAA catabolism, TCA flux, and CoA biosynthesis [60,88], the coordinated normalization of these pathways supports a mechanism whereby XFBD/GA primarily suppress the inflammatory axis, with metabolic reprogramming as a downstream consequence. Notably, these metabolomic alterations represent correlative evidence, and targeted metabolic flux analyses would be required to establish causal links between inflammatory pathway inhibition and specific metabolic changes. Importantly, the coordinated improvement of multiple core pathological features of MetS, particularly insulin resistance and lipid metabolic dysfunction, provides solid experimental evidence supporting further preclinical investigation of XFBD for specific metabolic disease endpoints.

To identify the key pharmacologically active constituents responsible for these effects, we employed an integrated network pharmacology–molecular docking–molecular dynamics strategy (Figure 8). From 91 identified chemical constituents of XFBD, PPI network analysis prioritized STAT3 and JUN as core protein targets. Molecular docking and molecular dynamics simulations revealed that GA, a triterpenoid saponin from *Glycyrrhizae Radix et Rhizoma*, formed the most stable binding complex with STAT3, maintaining a stable trajectory over 100 ns of MD simulation. In vivo validation confirmed that GA supplementation dose-dependently reduced hyperglycemia and suppressed both JNK and JAK/STAT pathway activation, largely recapitulating the anti-T2DM profile of the parent formula. However, it should be noted that GA likely represents only one of several bioactive constituents contributing to the overall antidiabetic effect of XFBD. Several other LC-MS-identified compounds—including uralsaponin C, ferulic acid, sinapic acid, and atractylenolide I—also exhibited favorable docking scores against JUN and/or STAT3 (Table 1), and may act synergistically or through complementary pathways. Given the multi-component nature of this herbal formula, the present study focused on GA as a representative and highly ranked active constituent; a systematic evaluation of the individual and combined contributions of other identified components will be pursued in our future work. GA represents a readily translatable lead compound for the development of therapeutics targeting insulin resistance and related metabolic disorders.

## 5. Conclusions and Limitations

This study demonstrates that XFBD dose-dependently ameliorates HSD-induced hyperglycemia, insulin resistance, glucolipid dysregulation and oxidative stress in *Drosophila*, primarily via inhibition of the JNK/JAK-STAT inflammatory axis rather than gut microbiota modulation. GA is identified as a key bioactive constituent that largely recapitulates the anti-diabetic and pathway-inhibitory effects of the complete formula. These findings expand the therapeutic scope of XFBD to T2DM and support further preclinical investigation into T2DM and NAFLD, two major clinical outcomes of metabolic syndrome.

Nevertheless, several limitations should be noted. The *Drosophila* model cannot fully recapitulate the pathological complexity of human T2DM, and mammalian validation is required to confirm translational potential, though the mechanistic insights gained from the *Drosophila* model may help refine subsequent mammalian studies in accordance with the 3R principles (Replacement, Reduction, Refinement). While our mechanistic conclusions are supported by pharmacological intervention combined with three complementary readouts (upd3-GFP, 10 × STAT-GFP reporters, and p-AKT Western blot), and are further corroborated by supplementary genetic and pharmacological evidence (upd3 overexpression, dominant-negative *bsk*, and MTX inhibition; Figure S5), definitive causal proof would require tissue-specific genetic epistasis experiments which are currently underway in our laboratory. The predicted binding of GA to STAT3 and JUN remains computationally derived and awaits biochemical verification via approaches such as SPR or CETSA. Additionally, GA is unlikely to be the sole bioactive component underlying XFBD’s anti-diabetic efficacy. Potential synergistic effects among other constituents of XFBD remain unexplored. Finally, the microbiota-independent mechanism proposed here, derived from fly experiments, requires validation in higher animal models. Collectively, future studies should also explore whether GA and other XFBD constituents act synergistically to enhance anti-inflammatory and insulin-sensitizing effects in mammalian models.

## Figures and Tables

**Table 1:** Binding energy of STAT3 and JUN with their corresponding active components.

Targets	Compounds	Binding Energy (kcal/mol)
	Glycyrrhizic acid	−9.5
	Uralsaponin C	−9.0
STAT3	3,4-Dihydroverbenalin	−6.8
	Atractylenolide I	−6.6
	Vulgarin	−5.9
	Ferulic acid	−5.6
	Sinapinic acid	−4.9
JUN	Glycyrrhizic acid	−7.8
	Uralsaponin C	−7.2
	Glabrolide	−7.1

## Data Availability

No new data were created or analyzed in this study. Data sharing is not applicable to this article.

## Supplementary Materials

The following supporting information can be downloaded at: https://www.mdpi.com/article/10.3390/biology15211975/s1, Figure S1. Changes in lactic *Lactobacillus* and *Acetobacter* loads upon XFBD intervention in HSD-induced *Drosophila*. Figure S2. Effect of XFBD on HSD-induced oxidative stress in *Drosophila*. Figure S3. Systematic prediction and verification of potential targets of XFBD via network pharmacology and molecular simulation. Figure S4. Effect of GA on hyperglycemia in HSD-induced *D. melanogaster* T2DM model. Figure S5. Genetic and pharmacological evidence for the causal role of the JNK/AP-1-upd3-JAK/STAT axis in HSD-induced insulin resistance in *Drosophila*. Table S1. Sequences of primers used in RT-qPCR. Table S2. Metabolites reversed by XFBD in the HSD-induced T2DM model.

## Abbreviations

The main abbreviations in this manuscript:

Akt	Protein kinase B
AMPK	Adenosine 5′-monophosphate-activated protein kinase
GA	Glycyrrhizic acid
HSD	High-sugar diet
IRS-1	Insulin receptor substrate 1
ND	Normal diet
PI3K	Phosphatidylinositol 3-kinase
STAT3	Signal transducer and activator of transcription 3
XFBD	Xuanfei Baidu Decoction
