# Supplementary material for: Xuanfei Baidu Decoction and Its Active Component Glycyrrhizic Acid Ameliorate Insulin Resistance in Type 2 Diabetes via JNK/JAK/STAT Axis Inhibition
Source: Biology (Basel). 2026 Nov 2;15(21):1975. doi: 10.3390/biology15211975 (PMC13761639; doi:10.3390/biology15211975)
Supplement: Supplementary file 1 [file biology-15-01975-s001.zip › supplementary figure.pdf]

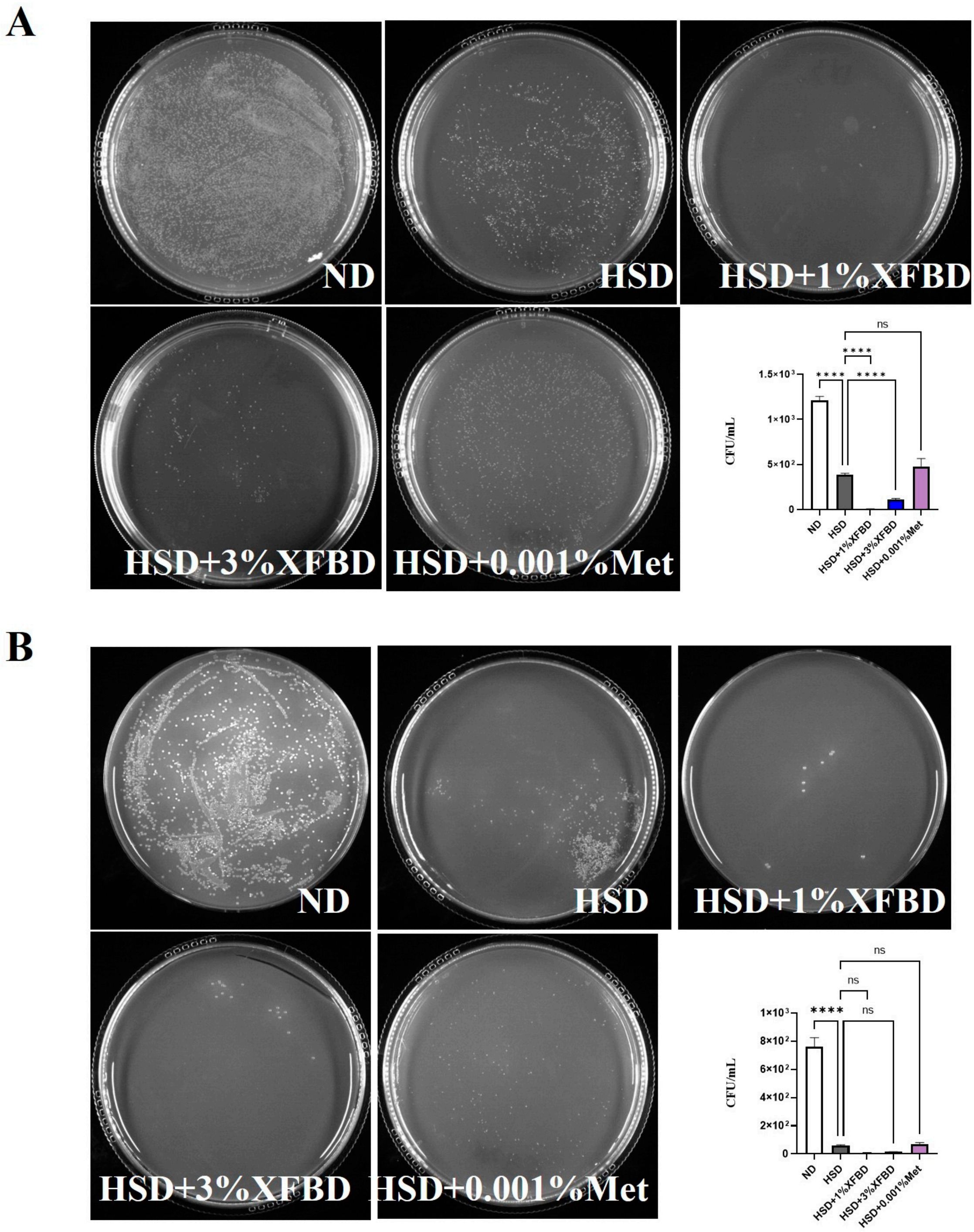

Figure S1 Changes in lactic *Lactobacillus* and *Acetobacter* loads upon XFBD intervention in HSD-induced *Drosophila*. (A) Plate culture results and statistical histogram of *Lactobacillus* loads (CFU/mL) in each group (n=4). (B) Plate culture results and statistical histogram of *Acetobacter* loads (CFU/mL) in each group (n=4). Data were analyzed using the t-test. (ns, no significance; *, $p < 0.05$; **, $p < 0.01$; ***, $p < 0.001$; ****, $p < 0.0001$).

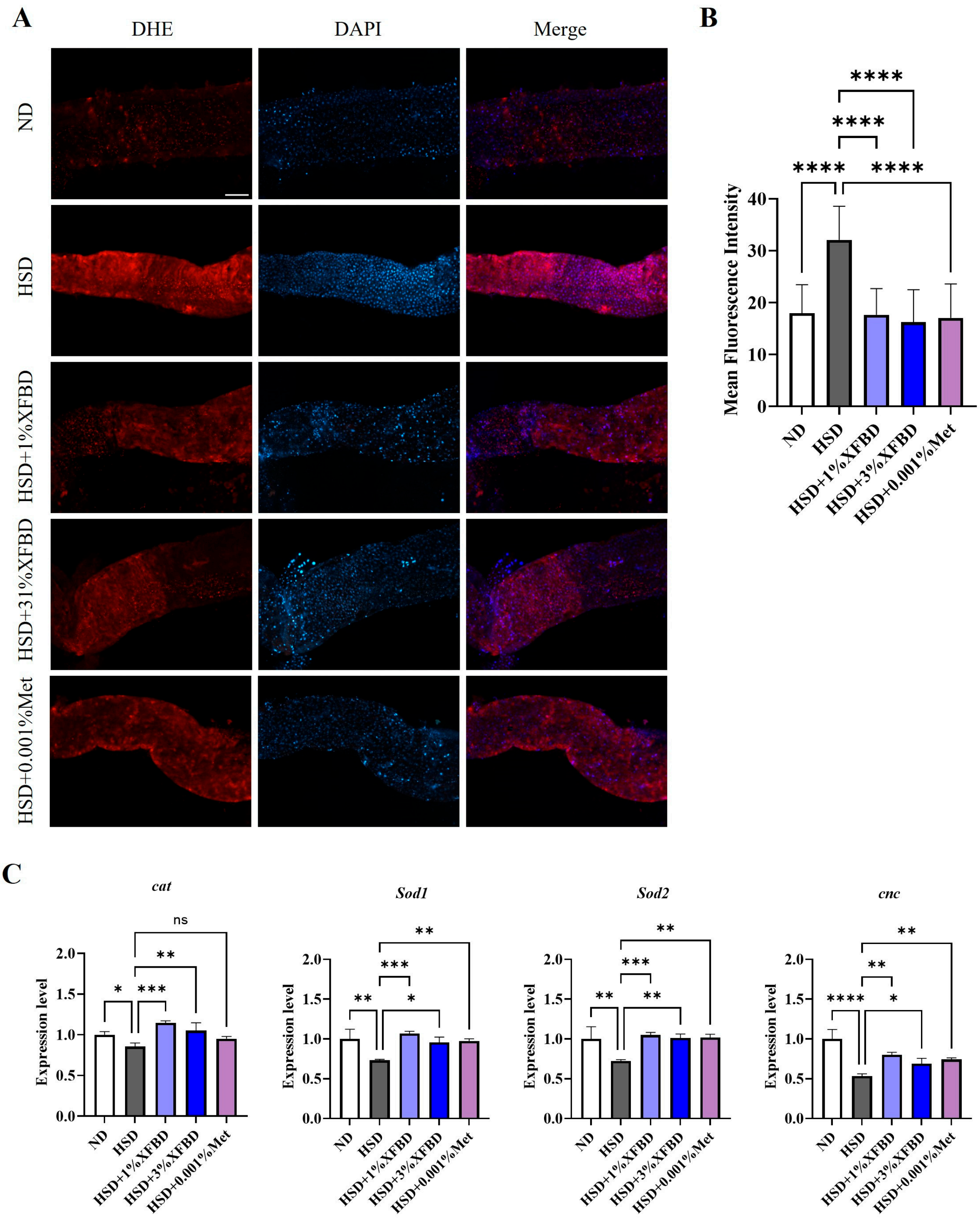

Figure S2 Effect of XFBD on HSD-induced oxidative stress in *Drosophila*. (A) Representative immunofluorescence images of DHE staining of D. melanogaster midgut across groups. DHE (red) labels intracellular superoxide (a major form of ROS); DAPI (blue) labels nuclei; and Merge indicates colocalization. Scale bar, 50 μm. (B) Quantitative

analysis of DHE fluorescence intensity. (C) Relative mRNA expression levels of antioxidant related genes. Data were analyzed by One-Way ANOVA (ns, no significance; *, $p < 0.05$; **, $p < 0.01$; ***, $p < 0.001$; ****, $p < 0.0001$).

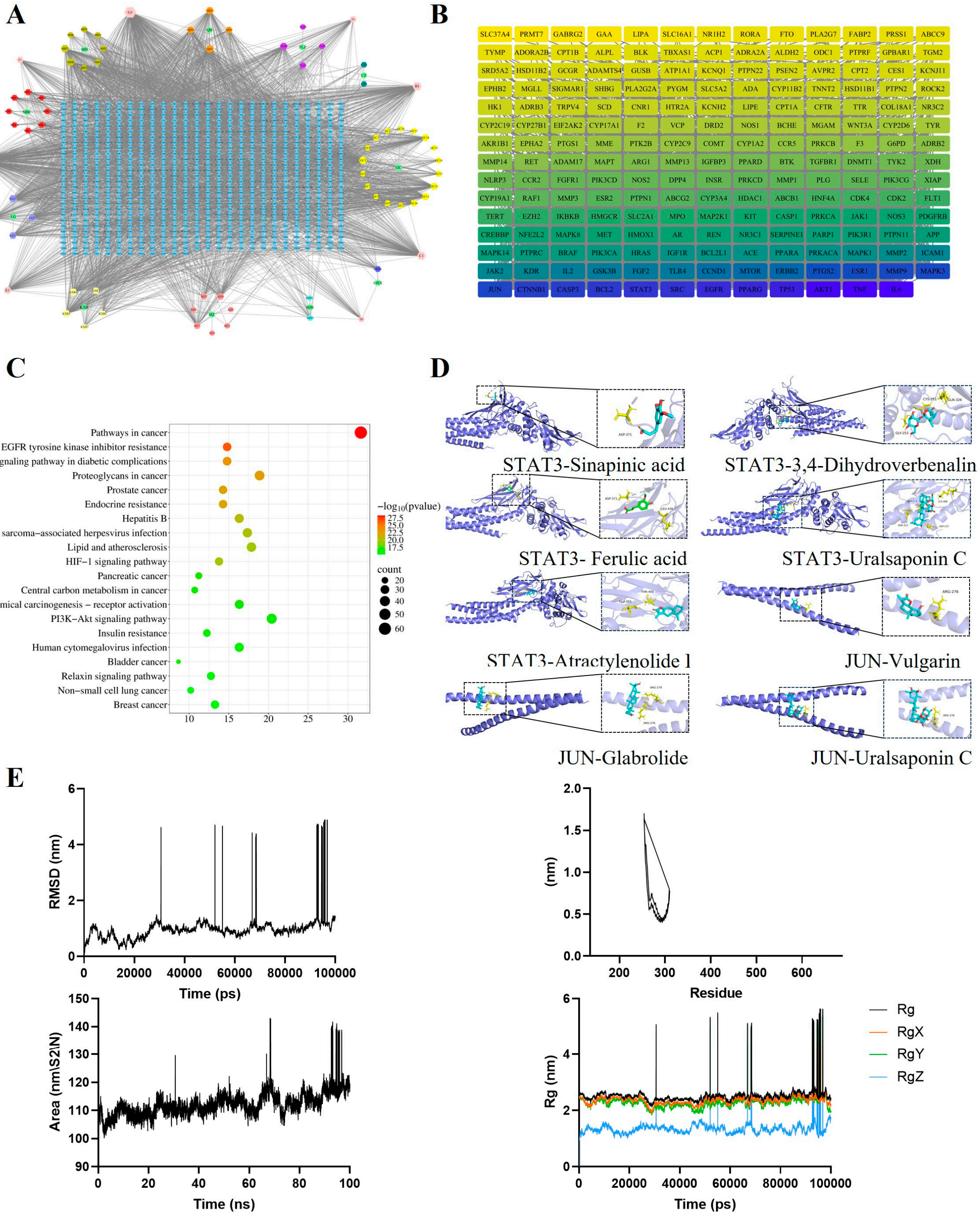

Figure S3 Systematic prediction and verification of potential targets of XFBD via network pharmacology and molecular simulation. (A) Herb-component target network. Round nodes: herbs; hexagonal nodes: active

components; diamond nodes: targets. The areas of hexagons and diamonds are proportional to their respective Degree values. Herb abbreviations: MH (*Ephedrae Herba*), QH (*Artemisiae Annuae Herba*), TLZ (*Descurainiae Semen*), CZ (*Atractylodis Rhizoma*), GC (*Glycyrrhizae Radix et Rhizoma*), GHX (*Pogostemonis Herba*), HJH (*Citri Grandis Exocarpium*), HZ (*Polygoni Cuspidati Rhizoma et Radix*), KXR (*Armeniacae Semen Amarum*), LG (*Phragmitis Rhizoma*), MBC (*Verbenae Herba*). Shared components: A1 (GHX/KXR), B1 (MH/KXR/CZ/HZ), C1 (GC/KXR), D1 (MBC/GHX/KXR), E1/E2 (HJH/HZ/GC), F1 (MBC/GC), G1 (HJH/CZ/HZ/GHX). (B) PPI network of potential targets of XFBD in treatment of T2DM. (C) KEGG pathway enrichment analysis of hub targets. (D) Molecular docking diagrams of STAT3 and JUN with other corresponding active components. (E) Molecular dynamics simulation of the JUN-GA complex. RMSD, RMSF, SASA, Rg.

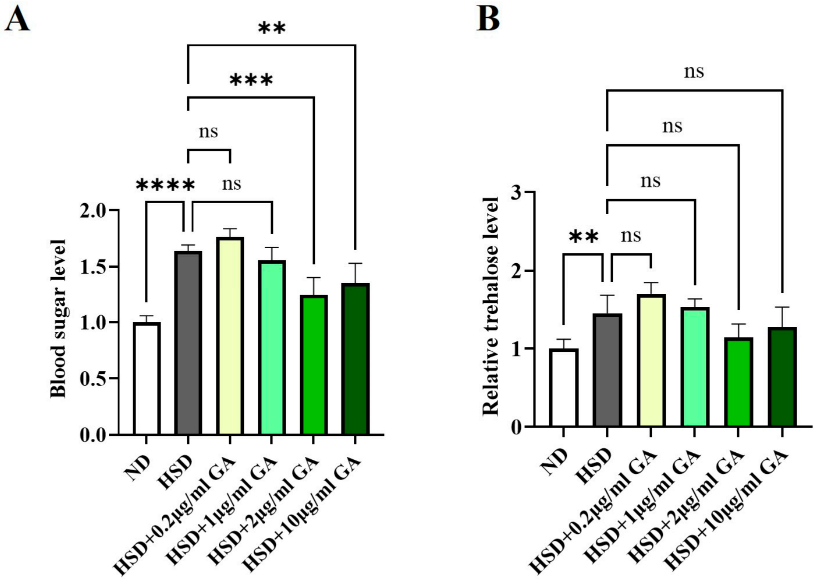

Figure S4 Effect of GA on hyperglycemia in HSD-induced *D. melanogaster* T2DM model. (A) relative total blood sugar level, and (B) trehalose level in flies of each group (n≥3). Data were analyzed by One-Way ANOVA (ns, no significance; *, $p < 0.05$; **, $p < 0.01$; ***, $p < 0.001$; ****, $p < 0.0001$).

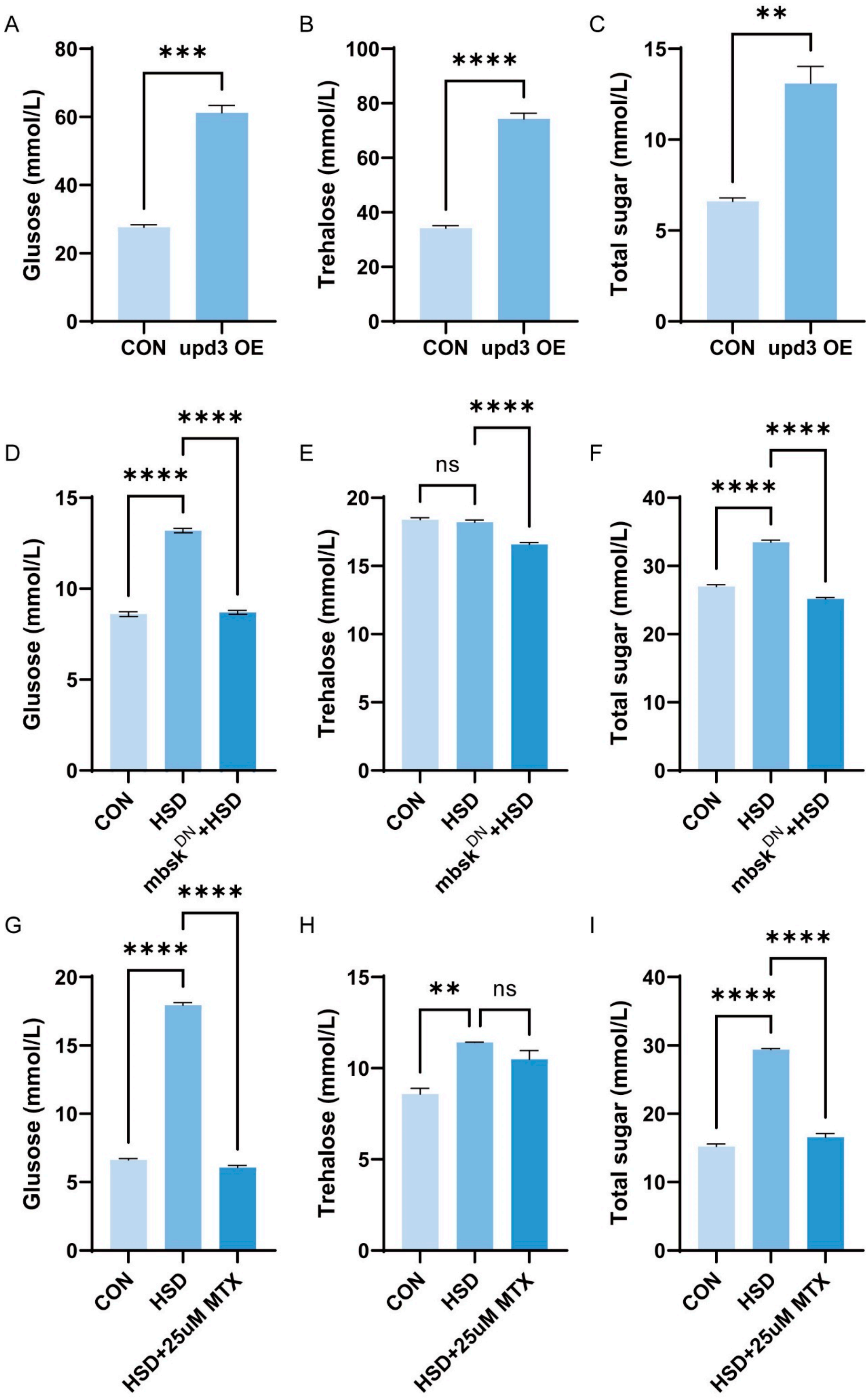
A
B
C
D
E
F
G
H
I
Glusose (mmol/L)
Trehalose (mmol/L)
Total sugar (mmol/L)
CON
upd3 OE
HSD
mbsk^{DN}+HSD
HSD+25uM MTX
***
****
**
ns

Figure S5. Genetic and pharmacological evidence for the causal role of the JNK/AP-1-upd3-JAK/STAT axis in HSD-induced insulin resistance in *Drosophila*. (A-C) *CG*-Gal4-driven overexpression of upd3 (upd3 OE) significantly increased hemolymph glucose (A), trehalose (B), and total sugar (C) levels compared with control (CON) flies. (D-F) Expression of dominant-negative basket ($mbsk^{DN}$), *Drosophila* JNK) under *MolyA*-Gal4 largely reversed HSD-induced elevation of hemolymph glucose (D), trehalose (E), and total sugar (F). (G-I) Pharmacological inhibition of JAK/STAT signaling with 25 µM Methotrexate (MTX) attenuated HSD-induced hyperglycemia, as shown by reduced hemolymph glucose (G), trehalose (H), and total sugar (I). Data represent mean ± SEM. Statistical significance was determined by one-way ANOVA with Tukey's post-hoc test. ns, not significant; $^{**}p < 0.01$; $^{***}p < 0.001$; $^{****}p < 0.0001$.
