# Supplementary material for: Xuanfei Baidu Decoction and Its Active Component Glycyrrhizic Acid Ameliorate Insulin Resistance in Type 2 Diabetes via JNK/JAK/STAT Axis Inhibition
Source: Biology (Basel). 2026 Nov 2;15(21):1975. doi: 10.3390/biology15211975 (PMC13761639; doi:10.3390/biology15211975)
Supplement: Supplementary file 1 [file biology-15-01975-s001.zip › Table S1 Sequences of primers used in RT.pdf]

Table S1 Sequences of primers used in RT-qPCR

Gene	Forward primer	Reverse primer
Dilp6	CGATGTATTTCCCAACAGTTTCG	AAATCGGTTACGTTCTGCAAGTC
4EBP	CTCCTGGAGGCACCAAACTTATC	TTCCCCTCAGCAAGCAACTG
InR	CTGGTGGTGCTGACAGAGAA	GCAGCTGACAACTGGCATTA
Meep	CTCTCGGAACTGAAAAGAG	AACTGGGAGTCCCTTAAATC
Nlaz	GGACAACCCTCGAATGTAACT	GACGGCGTGATGACTCGTAATC
Tret	CTCTGCAGCATTCATTGTGGG	GTCAGAAGCATGGCGATGTC
Glut3	TGTGTGGTGCCCTGATCTTT	GAGGAAAACATGCAGCAGCGG
Pepck	TCATCATCACCCAAGAGCA	CCACCACATAGGGCAGAGT
G6Pase	ATCAATCTCTCTGGGTGGC	TGTTGCTGTAGTAGTCGGTGTCC
Tps	GAGCTATGGTTGACAAGGCG	ACGACCCTTATTCCACTGCA
Lsd-1	TTGGTTTCGGCCCTGAATGA	GGGAGAAGCGTTGACCATGA
cat	ACCAGGGCATCAAGAATCTG	AACTTCTTGGCCTGCTCGTA
Sod1	GTTCGGTGACAACACCAATG	GGAGTCGGTGATGTTGACCT
Sod2	TGTTTCGTGGCCGTAAAAT	GTCTGGTGCTTCTGGTG
cnc	GATACCGCCTCGCTGGACCC	GATCCTGCAGCGCATGTGGC
Impl2	CTGGCTCGAATTCCACCAAGAC	ATCTCGCAAACGATCTCGATG
Socs36E	CTGGGGTGCTATTGATTTGGAGT	TCGAGGATGGGATGTGGAC
TotA	TTCCGACGAAGATCGTGAGG	CTGGGGTGCTATTGATTTGGAGT
upd1	CCACGTAAGTTTGCATGTTG	CTAAACAGTAGCCAGGACTC
upd2	AGTGCGGTGAAGCTAAAGACTTG	GCCCGTCCCAGATATGAGAA
upd3	ACAGATTCCTGCCCCGTCT	GGTCGCGATGGCGT
mmp1	TGGTTTGTTTTCGCCCCATA	GAGATGCCCACTTTGACGAC
mmp2	TACTTGTGGCGCATTGGAAC	ATCGATGTGGGTCAAAGTGG
