# Supplementary material for: Xuanfei Baidu Decoction and Its Active Component Glycyrrhizic Acid Ameliorate Insulin Resistance in Type 2 Diabetes via JNK/JAK/STAT Axis Inhibition
Source: Biology (Basel). 2026 Nov 2;15(21):1975. doi: 10.3390/biology15211975 (PMC13761639; doi:10.3390/biology15211975)
Supplement: Supplementary file 1 [file biology-15-01975-s001.zip › WB Original Images for Blots or Gels .pdf]

1.

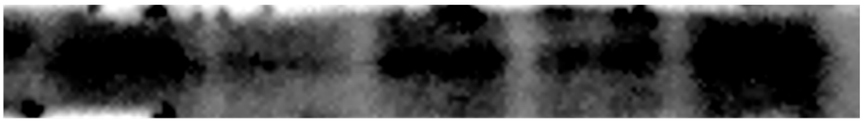

PAKT 60KD

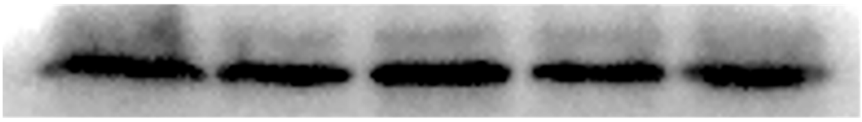

AKT 60KD

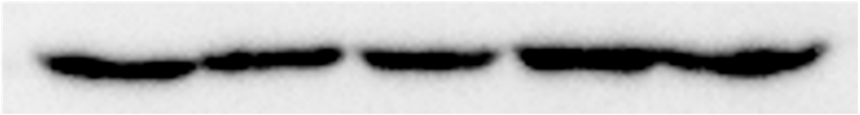

Actin 43KD

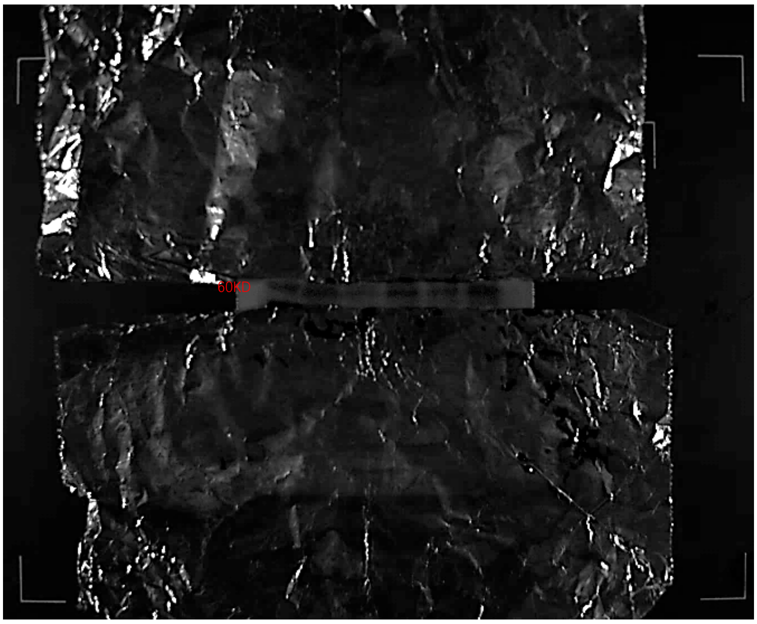

PAKT

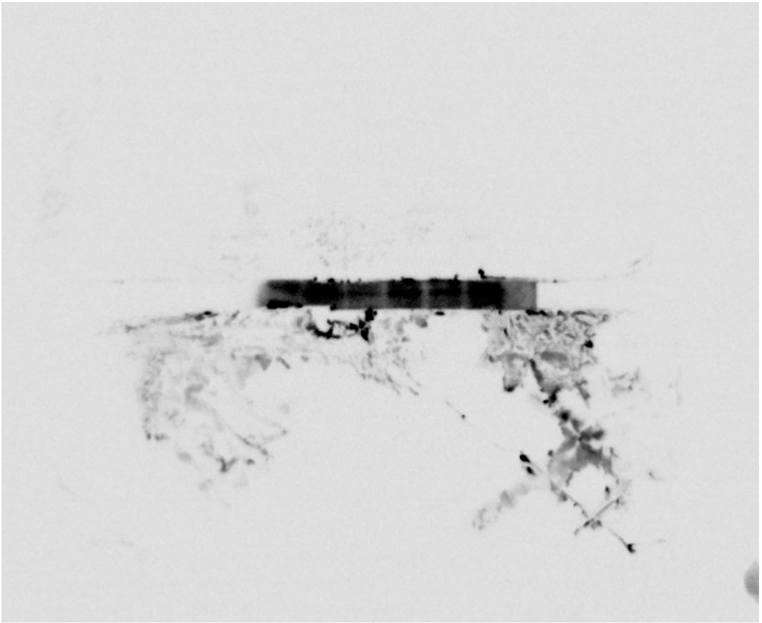

PAKT

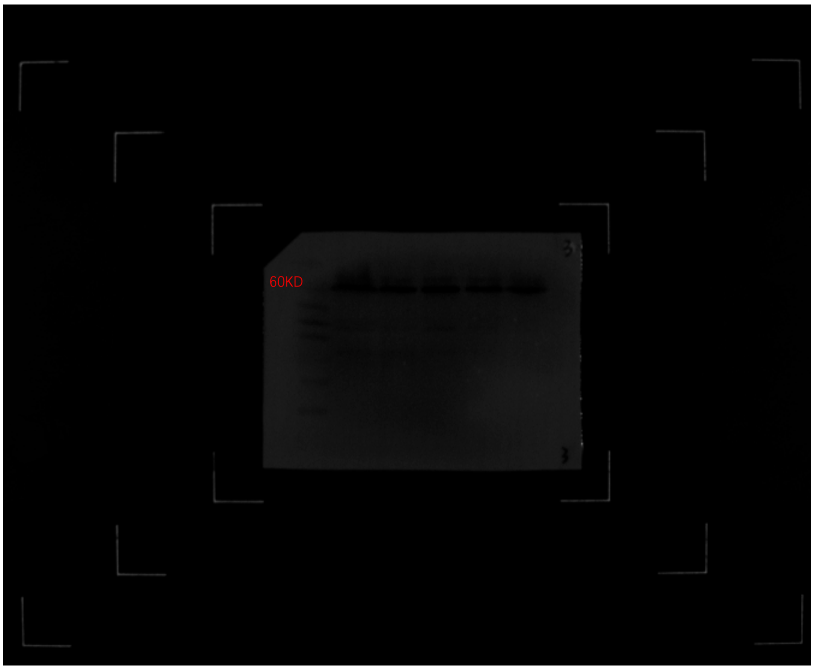

AKT

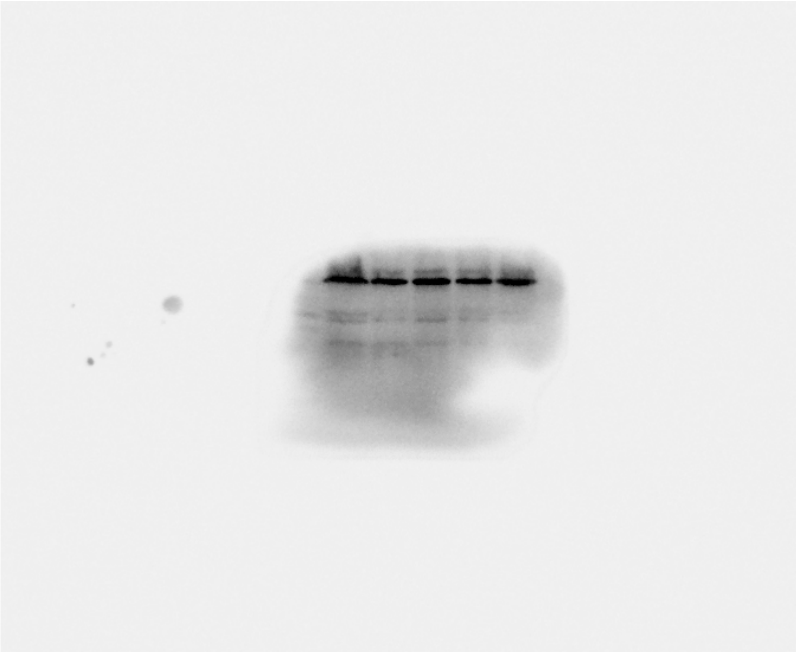

AKT

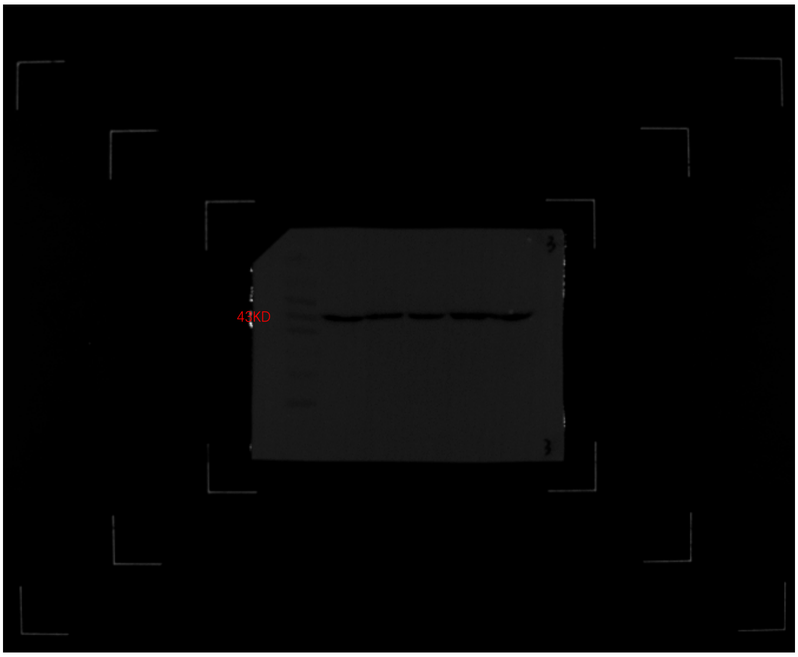

Actin

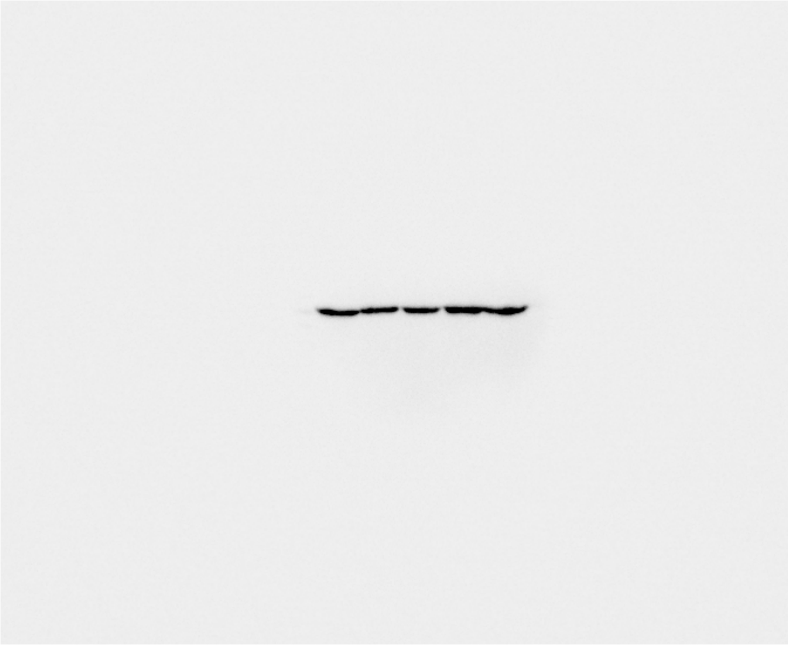

Actin

2.

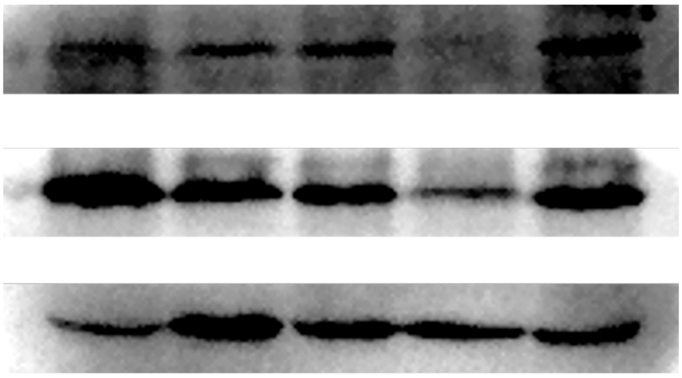

PAKT 60KD

AKT 60KD

Actin 43KD

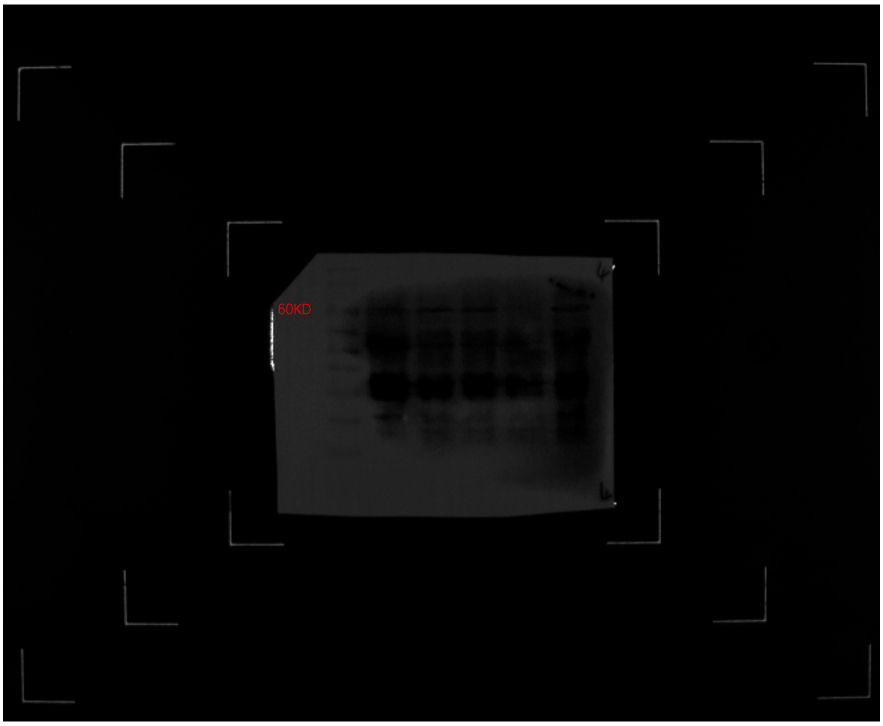

PAKT

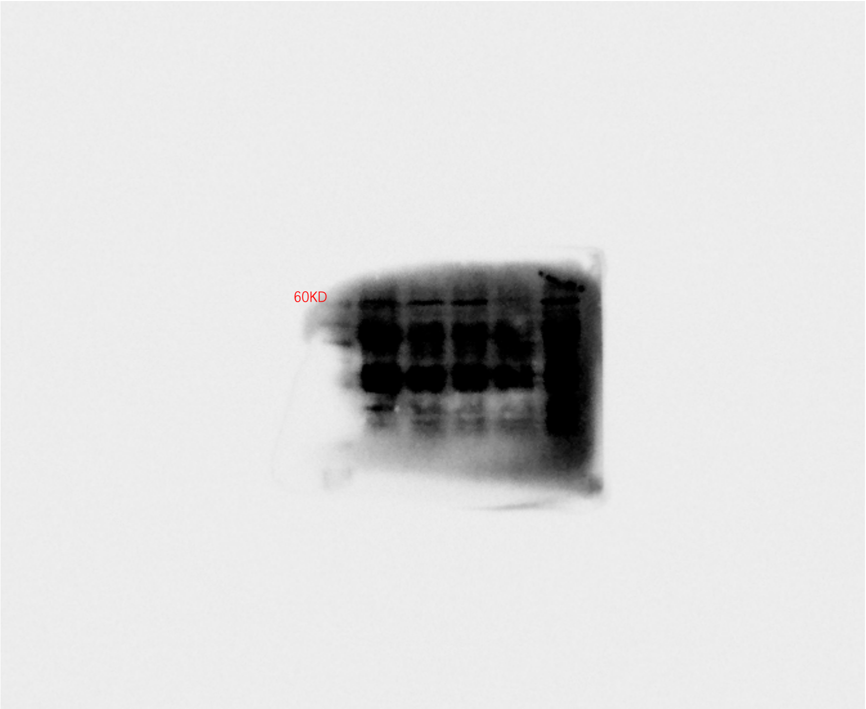

PAKT

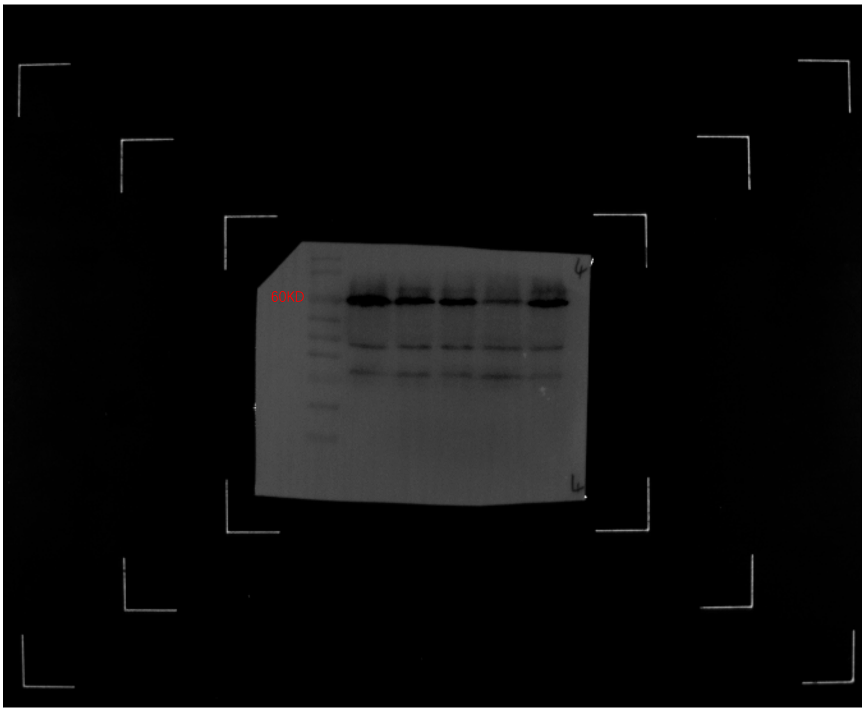

AKT

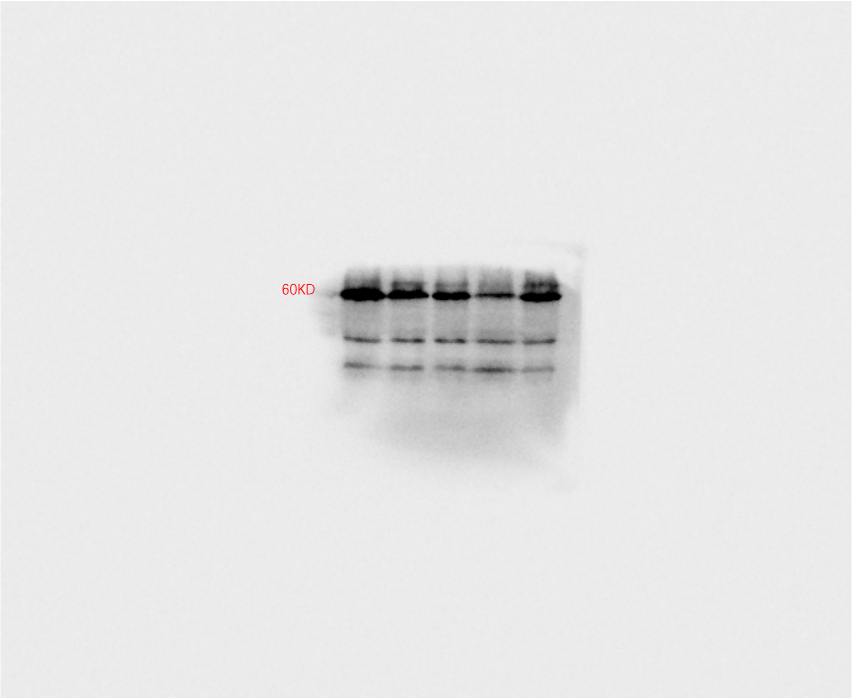

AKT

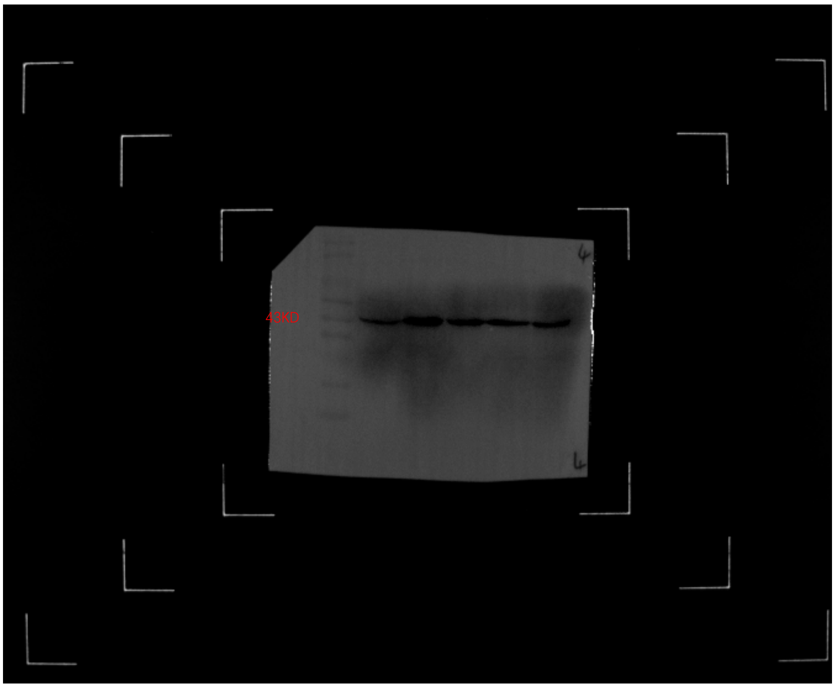

Actin

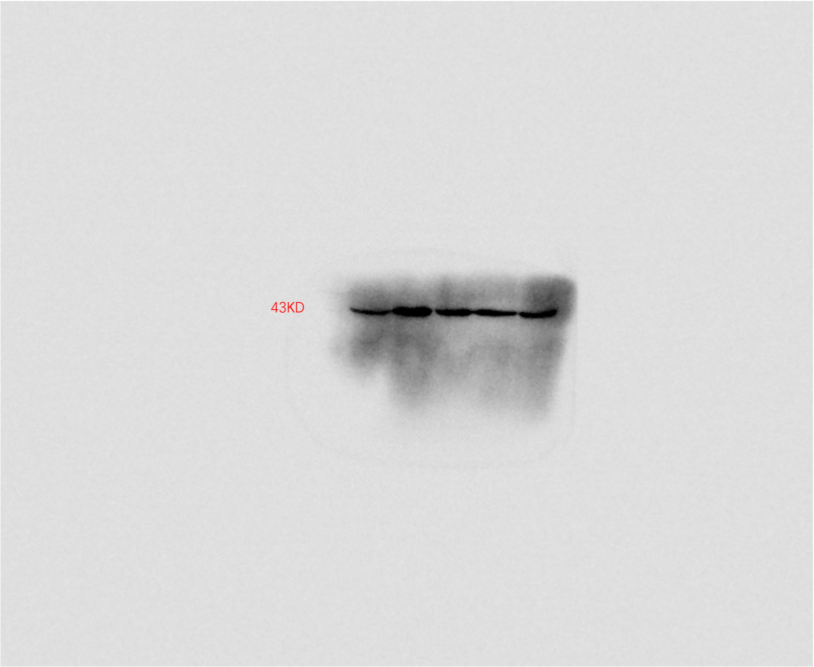

Actin

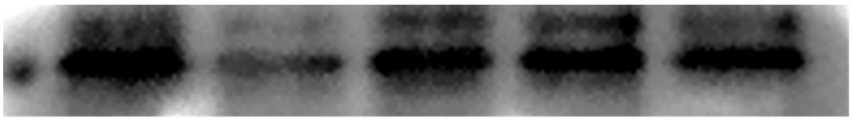

PAKT 60KD

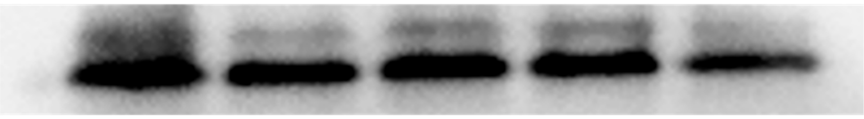

AKT 60KD

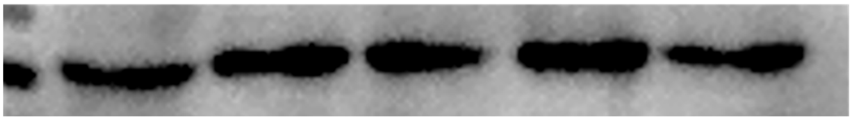

Actin 43KD

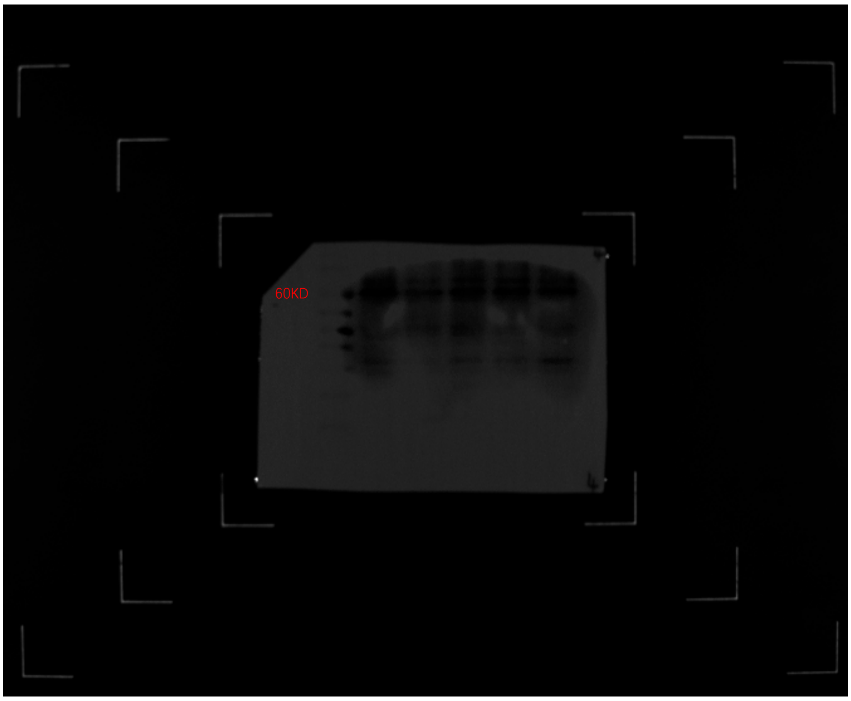

PAKT

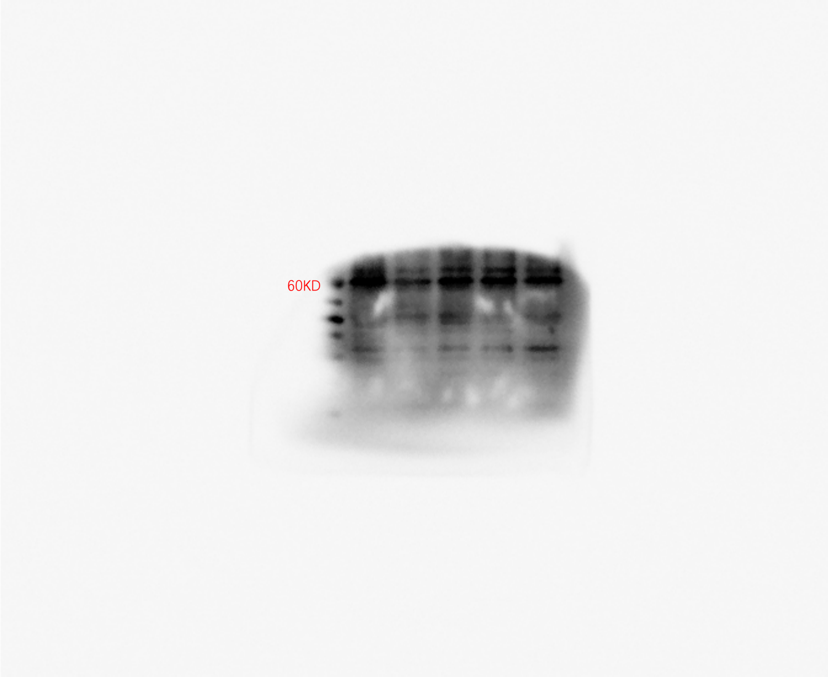

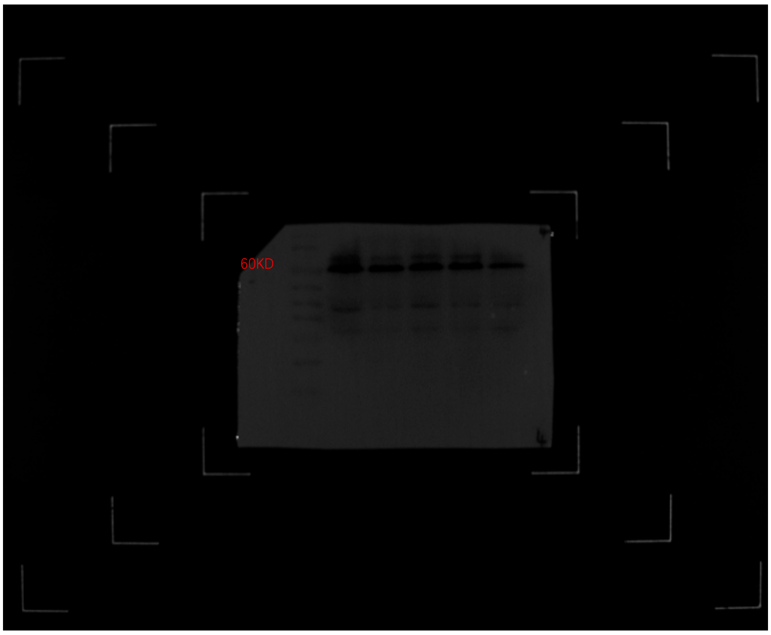

AKT

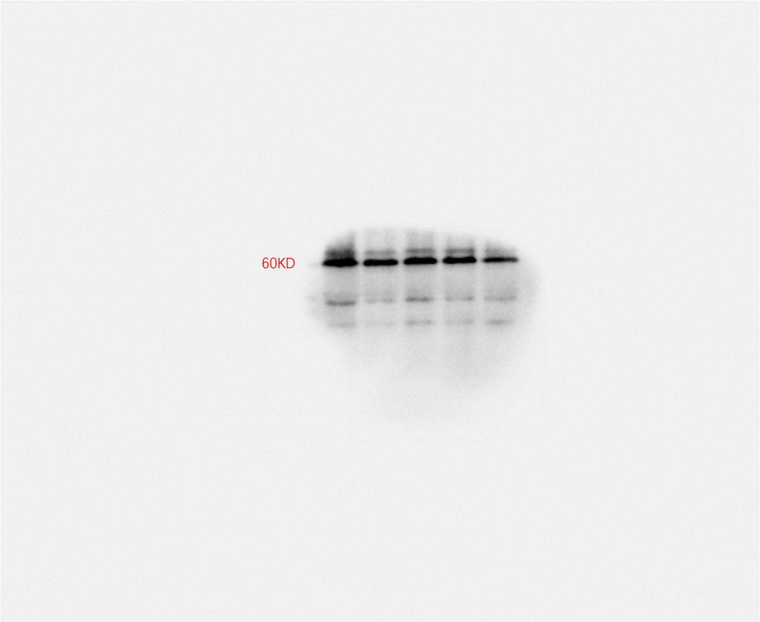

AKT

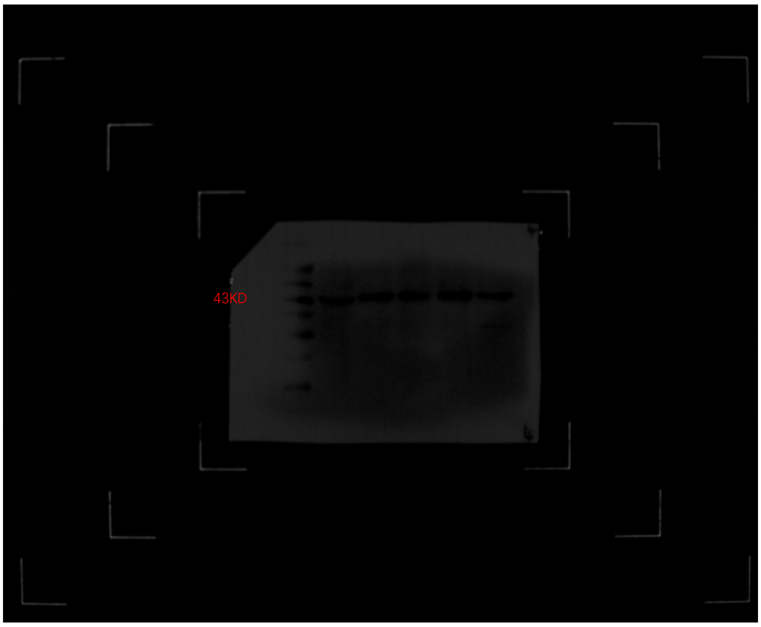

Actin

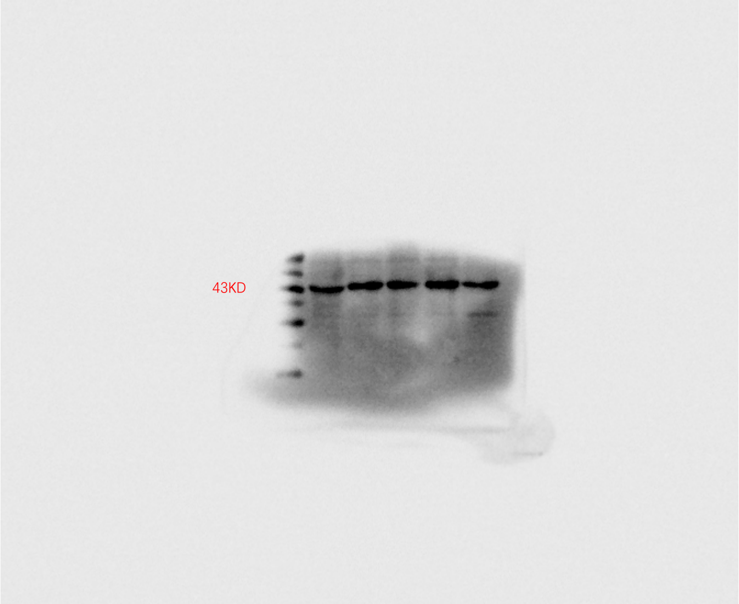

Actin
